# Coherence Between Brain Activation and Speech Envelope at Word and Sentence Levels Showed Age-Related Differences in Low Frequency Bands

**DOI:** 10.1162/nol_a_00033

**Published:** 2021-05-07

**Authors:** Orsolya B. Kolozsvári, Weiyong Xu, Georgia Gerike, Tiina Parviainen, Lea Nieminen, Aude Noiray, Jarmo A. Hämäläinen

**Affiliations:** Department of Psychology, University of Jyväskylä, Finland; Centre for Interdisciplinary Brain Research (CIBR), University of Jyväskylä, Finland; Niilo Mäki Institute, Jyväskylä, Finland; Centre for Applied Language Studies, University of Jyväskylä, Finland; Laboratory for Oral Language Acquisition (LOLA), University of Potsdam, Germany

**Keywords:** speech perception, development, magnetoencephalography, speech tracking, coherence, auditory responses

## Abstract

Speech perception is dynamic and shows changes across development. In parallel, functional differences in brain development over time have been well documented and these differences may interact with changes in speech perception during infancy and childhood. Further, there is evidence that the two hemispheres contribute unequally to speech segmentation at the sentence and phonemic levels. To disentangle those contributions, we studied the cortical tracking of various sized units of speech that are crucial for spoken language processing in children (4.7–9.3 years old, *N* = 34) and adults (*N* = 19). We measured participants’ magnetoencephalogram (MEG) responses to syllables, words, and sentences, calculated the coherence between the speech signal and MEG responses at the level of words and sentences, and further examined auditory evoked responses to syllables. Age-related differences were found for coherence values at the delta and theta frequency bands. Both frequency bands showed an effect of stimulus type, although this was attributed to the length of the stimulus and not the linguistic unit size. There was no difference between hemispheres at the source level either in coherence values for word or sentence processing or in evoked response to syllables. Results highlight the importance of the lower frequencies for speech tracking in the brain across different lexical units. Further, stimulus length affects the speech–brain associations suggesting methodological approaches should be selected carefully when studying speech envelope processing at the neural level. Speech tracking in the brain seems decoupled from more general maturation of the auditory cortex.

## INTRODUCTION

Brain structure and function continue to develop into early adulthood, with some evidence for different trajectories for the left and right hemispheres (Gogtay et al., 2004; Pang & Taylor, 2000; Parviainen et al., 2019). In adults, important functional differences between the left and right hemispheres have been demonstrated when processing syllable and phonemic information (e.g., Poeppel, 2014). However, little is known about the development of this functional specialization in children. Functional near-infrared spectroscopy (fNIRS) and magnetic resonance imaging (MRI) have provided evidence for a significant leftward asymmetry for speech processing that is already present from birth (Dehaene-Lambertz et al., 2002; Pena et al., 2003). Drawing on these findings, we used magnetoencephalography (MEG) to examine how hemispheric specialization is reflected in brain responses to various speech units (sentence, words, syllables) and to uncover whether this specialization differs between children and adults. To achieve those goals, we combined two experimental approaches: examining general indices of auditory maturation as reflected in the age-related changes of onset-responses (event-related fields [ERF]) to simple speech sounds alongside examination of word and sentence tracking in different frequency bands, as measured by coherence.

Previously, long lasting maturational effects have often been studied using the event-related potentials (ERPs) and their magnetic equivalent ERFs to short sounds with EEG and MEG. The auditory ERPs in infancy and in the preschool age show prominent P1 and N2 responses, which as children enter childhood start to become earlier in latency and decrease in amplitude. Additionally, P1 and N2 responses are separated by emerging N1 and P2 responses around the age of 8 to 9 years (Albrecht et al., 2000; Ponton et al., 2000).

Differences in hemispheric maturation rates have also been observed using ERFs. The N1m patterns measured with MEG were more adult-like in 7- to 8-year-olds in the right hemisphere than in the left (Parviainen et al., 2019). This suggests fine-grained developmental trajectories of the different auditory regions with clearly immature patterns of activation in the auditory cortex around early school age (8 to 9 years old).

While studying the event-related potentials and fields in response to individual phonemes and syllables is a useful method to investigate the well-known maturational effects of auditory processing, auditory information in speech spans across multiple timescales encompassing phonemes, syllables, words, and phrases. Multi-time resolution models of speech processing (Ghitza, 2011; Ghitza & Greenberg, 2009; Poeppel, 2003; Poeppel & Assaneo, 2020) propose that speech information is processed and integrated in a hierarchical and interdependent manner by phase alignment or neural entrainment of the involved oscillatory networks in the auditory cortices with different specialization for the left and right auditory areas.

Coherence analysis can be used to study speech perception in these longer speech segments. Coherence is the computation of synchrony between two signals in the frequency domain. The coherence value reflects the consistency of phase difference between two signals (here between the speech envelope and brain activity) at any given frequency. This technique can be used to investigate tracking of the speech signal in the brain, which has been argued to reflect relevant linguistic operations such as parsing and chunking of hierarchical linguistic structures of speech (Bourguignon et al., 2013; Ding et al., 2016; Gross et al., 2013; Molinaro & Lizarazu, 2018; Peelle & Davis, 2012).

Neuronal oscillations in frequency bands present in speech (delta, 1–3 Hz, theta, 4–8 Hz, beta, 15–30 Hz, and low gamma, 30–50 Hz; Poeppel, 2014) have been theorised to provide a basis for parsing the continuous speech signal into different linguistic units (e.g., delta: syllable stress patterns; theta: syllables; beta: onset-rime units; low gamma: phonetic information; Ghitza et al., 2013; Leong & Goswami, 2014; Poeppel, 2014; Poeppel & Assaneo, 2020). In this framework, the linguistic information associated with the different timescales would be then integrated to give the final speech percept. Low frequency cortical activity appears to synchronise to the rhythms of multiple linguistic units (Ding et al., 2016, 2017), while higher frequencies (such as beta and gamma) may be more sensitive to syntactic and semantic information (Ding et al., 2016). Together, these results suggest that during listening to connected speech, the brain synchronizes cortical rhythms to track the rhythm of the different linguistic units (Ding et al., 2017).

Speech processing involves both left and right auditory cortices (Poeppel, 2003; Poeppel & Assaneo, 2020). In its early stage the representation of the input speech signal has a bilateral symmetry, which then branches out in subsequent processing steps. Left auditory areas have been suggested to sample information from short (20–40 ms) integration windows (Giraud et al., 2007; Poeppel, 2003; Poeppel & Assaneo, 2020), and right areas to sample information from longer (150–200 ms) integration windows (Giraud et al., 2007; Luo & Poeppel, 2007; Poeppel, 2003; Poeppel & Assaneo, 2020). These differences are reflected in oscillatory neuronal activity in different bands (mostly in gamma and theta bands, respectively).

However, changes in brain activity have been reported during childhood with respect to general auditory sound processing as well as more specific speech processing (e.g., Ríos-López et al., 2020; Uhlhaas et al., 2010). Developmental changes in neural synchrony have been demonstrated (for a review, see Uhlhaas et al., 2010) using auditory stimulation (Müller et al., 2009), whereby young children showed reduced synchronisation in the delta and theta (Müller et al., 2009) frequencies compared to adolescents and adults.

There is converging evidence that hemispheric specialisation to different windows of integration for auditory information and speech is present from the first year of life; however, results differ as to which hemisphere shows the strongest response to long speech-like chunks (Telkemeyer et al., 2009, 2011). The developmental pattern of hemispheric dominance for processing spoken sentences seems to shift between brain hemispheres with age. Greater entrainment to speech was found in the left hemisphere compared to right in the theta band with 7-month-old infants (Kalashnikova et al., 2018). However, this specialization was not found in young children between the ages of 4 and 7 years (Ríos-López et al., 2020) in the delta band. Finally, a higher correlation in the right as compared to the left hemisphere between the amplitude envelope of sentences and their corresponding brain responses was found in older 9- to 13-year-old children (Abrams et al., 2008, 2009).

Building on those findings, the current study investigated (a) age-related differences and (b) hemispheric balance in word and sentence tracking in low frequency bands to separate the word to phrasal levels of processing. Based on previous studies on adults and older children, we expected hemispheric differences to already be present in 5- to 9-year-olds in the delta (1–4 Hz) and theta (4–8 Hz) bands with the right hemisphere showing higher coherence than the left hemisphere.

To examine if and how the maturation of the synchrony measures is related to the established maturation of the onset response (reflected in the changes in ERFs to syllables), we compared the coherence values for words and sentences with the age-related changes in the N1m response to syllables. Evoked brain activity to sounds has been shown to change from preschool to school age and to adulthood. While the specific N1m response is absent in early childhood, it seems to emerge at around 8 to 9 years of age and only become fully mature in adulthood (Albrecht et al., 2000; Ponton et al., 2000). If the N1m amplitude has a common underlying maturational mechanism with the speech tracking index, our results should show similar developmental effects. On the other hand, synchronization of brain activity to speech could utilize partly separate brain mechanisms that follow a different developmental trajectory and are affected more by environmental input than by developmental changes reflected by N1m.

We also examined correlations between the processing of speech envelopes and phonological skills. Speech envelope processing has been related to segmentation into syllable and phoneme level elements (Poeppel, 2014). As for phonological skills, broadly defined they include the awareness of various speech units (e.g., phonemes, syllables, words), working memory operations for speech sounds, and access to phonological representations (e.g., Fowler, 1991; Goswami & Bryant, 2016; for a review, see Noiray, Popescu et al., 2019). These are thought to be represented, for example, by rapid naming, phoneme deletion, and speech repetition tasks. Based on this, we hypothesized that speech envelope processing could be linked to phonological skill development (Goswami, 2011).

## MATERIALS AND METHODS

### Participants

Two age groups participated in the study: typically developing children and young adults. The adults were studying at the University of Jyväskylä, Finland. Table 1 shows the number of participants, mean age and age-range, gender, handedness, and average hearing level for each group. All participants were Finnish native speakers.

**Table 1.** Description of participants

	**Children**	**Adults**
# of participants included in the analysis (measured in MEG)	34 (47)	19 (19)
Mean age (*SD*)	7.53 (1.34)	24.80 (3.73)
Age range (Minimum–Maximum; y = years, m = months)	4y8m–9y4m	20y3m–35y2m
Gender ratio (M:F)	18:16	2:17
Handedness (left:both:right)	5:1:28	0:1:18
Average hearing level in DBs (left:right ear)	21.25:21.37	Self-report of normal hearing level

The children were recruited via the National Registry of Finland and the adults via email lists of the university. Exclusion criteria at the time of recruitment were head injuries, ADHD or learning difficulties, neurological diseases and medication affecting the central nervous system, or any reported hearing deficits. Children recruited for the study were typically developing and did not present any neurological, cognitive, or language-related deficiency. In addition, the hearing level of the participants was tested using audiometry, with most of them performing at or below 25 dBs for 250 Hz, 500 Hz, 1000 Hz, and 2000 Hz sounds in the left and right ears.

After data collection 13 participants were excluded overall, all of them from the child group. Five were excluded based on the measurement because of too much movement and inability to follow instructions during the recording, two because of noisy data, four because of technical problems (instrumentation failure or software issues), one based on incidental findings during the measurements (based on the neurologist’s report), and one because of high amplitude fluctuations in the data.

Enrolment in the study was voluntary; all adults and children participants as well as their parent/caregiver provided written informed consent prior to their participation in the study. Subsequent to the MEG study, all participants received either a movie ticket or a gift card as compensation for their participation. Individual structural MR images were acquired from a private company offering MRI services (Synlab Jyväskylä). T1-weighted 3D-SE images were collected on a GE 1.5 T (GoldSeal Signa HDxt) MRI scanner using a standard head coil and with the following parameters: TR/TE = 540/10 ms, flip angle = 90, matrix size = 256 × 256, slice thickness = 1.2 mm, sagittal orientation.

This study was carried out in accordance with the Declaration of Helsinki and approved by the Ethical Committee of the University of Jyväskylä, Finland.

### Behavioural Test Battery

First, we conducted a battery of behavioural tests assessing the children’s general cognitive abilities, with an emphasis on language-related skills. For a description of the behavioural tests, see Table 2.

**Table 2.** Description of behavioural test scores

**Behavioural measure**	**Subtest**	**Mean (*SD*) score reported**	**Children**		**Adults**
WPPSI-III, WISC-IV, WAIS-IV	Block design	sp	10.00 (3.82)	10.96 (3.15)	11.26 (3.21)
	Vocabulary	sp	11.36 (3.01)	10.46 (3.07)	13.79 (2.02)
	Digit span	sp	x	9.96 (2.15)	11.26 (2.96)
NEPSY	Repetition of nonsense words	sp	10.09 (2.43)		x
	Oro-motor task	sp	10.30 (2.81)		x
NEPSY-II	Visuo-motor task (car and motorcycle)	combined sp	9.42 (3.02)		x
	Phonological processing	sp	11.33 (2.33)		x
	Repetition of sentences	# correct	25.62 (3.08)		x
Rapid automatized naming (RAN)	Objects	time (s)	64.25 (13.66)		34.37 (7.78)
	Letters	time (s)	34.70 (10.64)		18.90 (4.62)
Letter knowledge task		total	22.67 (8.27)		x
Lukilasse	Word reading	percentile	46.38 (40.32)		x
Pseudoword list reading		total	34.45 (13.98)		x
Pseudoword text reading		fluency	99.59 (0.22)		x
Lukilasse	Dictation	percentile	58.10 (39.06)		x

*Note*. sp: standard point; *SD*: standard deviation; WPPSI-III: Wechsler Preschool and Primary Scale of Intelligence (Wechsler, 2003a); WISC-IV: Wechsler Intelligence Scale for Children (Wechsler, 2003b); WAIS-IV: Wechsler Adult Intelligence Scale (Wechsler, 2008); NEPSY: Neuropsychological Assessment test battery I (Korkman et al., 1998); NEPSY II (Korkman et al., 2008); RAN: Rapid automatized naming (Denckla & Rudel, 1976); Lukilasse (Häyrinen et al., 1999).

Three different age-appropriate tests, WPPSI-III (Wechsler, 2003a), WISC-IV (Wechsler, 2003b), and WAIS-IV (Wechsler, 2008), were used to measure participants’ visuo-spatial reasoning and vocabulary, and two tests, WISC-IV and WAIS-IV, were used for working memory. The motor development of the participants was tested using subtests from the Developmental Neuropsychological Assessment (NEPSY; Korkman et al., 1998), the oro-motor task, and the NEPSY II visuo-motor task (Korkman et al., 2008). Participants’ phonological processing was tested using the NEPSY II subtest. To assess speed of lexical retrieval, the Rapid automatized naming (RAN; Denckla & Rudel, 1976) Objects and Letters subtests were used. To measure memory for sentences, the NEPSY II Sentence Repetition subtest was used.

Reading skills were tested using the word reading task from the Lukilasse test battery (Häyrinen et al., 1999), the pseudoword reading task adapted from TOWRE (Torgesen et al., 1999), and the pseudoword text reading task (Eklund et al., 2015).

For a detailed description of the behavioural tests, see Supplementary Material 1 (supporting information can be found online at https://www.mitpressjournals.org/doi/suppl/10.1162/nol_a_00033).

### Stimuli

Three types of stimuli characterizing various temporal and linguistic structures were used for the speech tracking task: syllables, words, and sentences. Syllables varied in consonants’ place of articulation (moving from front to back: bilabial stop /p/, dental stop /t/, and palatal stop /k/), while the vowel remained identical (/a/).

Each syllable was presented 18 times (total of 54 syllable presentations), and words starting with the same syllables (18 words for each syllable, total of 54 words), as well as 54 sentences, each starting with one of the word category stimuli. For a description and exemplars of the stimuli, see Table 3.

**Table 3.** Description of stimuli

**Stimulus type**	**Average duration (ms)**	***SD***	**Range (ms)**	**Exemplars: Finnish**	**English translation**
Syllable	209.33	25.58	185–236	ka, ta, pa	
Word	574.54	103.22	352–797	kala, paju, talo	*fish*, *willow*, *house*
Sentence	1,438.54	240.49	1,039–2,051	Kala on akvaariossa. Paju on taipuisa puu. Talo on aivan uusi.	*The fish is in the aquarium. A willow is a flexible tree. The house is brand new.*

All words were common, everyday nouns. The words were 2 to 3 syllables long. Sentences were composed of 3 to 4 words and always started with a noun followed by a form of the verb “to be” in the present tense. Stimuli were chosen with the help of an expert developmental linguist. The stimuli were produced by a female native Finnish speaker. All stimuli were separate, unique tokens produced separately. Stimuli were recorded using a 44 kHz sampling frequency, 32-bit quantisation in a professional recording studio. The sound files were cut into individual segments using Praat (Boersma & Weenink, 2018).

The same syllables and words were used for each stimulus type to get comparable onset evoked brain responses. To see the list of stimuli used, see Supplementary Material 2.

### Procedure

#### Experimental design

**Figure 1.**
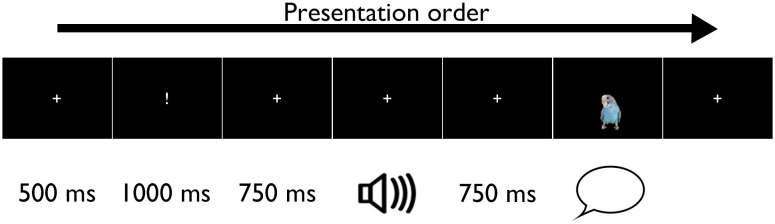
Schematic representation of one trial of the experimental paradigm. Data analysis was focused on the time-window during the stimuli presentation, indicated by the picture of the loudspeaker.

Each speech tracking trial consisted of a fixation cross in the middle of the screen for 500 ms, then an exclamation mark appeared in the same space for 1,000 ms signalling that a sound is going to come soon, followed by the fixation cross for 750 ms. The auditory stimuli were then presented via earphones, with the fixation cross on the screen. The fixation cross remained on the screen for 750 ms after the end of sound. This was followed by a still image of a parrot appearing for 1,250–4,250 ms (presentation duration depended on the type of stimuli heard) which provided the cue for the participants to repeat the previously heard stimuli aloud (see Figure 1).

Participants were instructed to first listen to a speech sequence (i.e., a syllable, a word, or a sentence) and to repeat it after seeing the visual cue on the screen (a parakeet). The visual stimuli were presented on a black background with white standard characters (a cross for fixation and an exclamation mark alerting to the auditory stimuli) in Times New Roman font and a font size 64. The bird stimuli were 9 × 15 cm in size on the projection screen. Here only the time-window of the auditory stimulus presentation was analysed.

Participants were first given instructions and 6 practice trials (2 of each type of stimuli, presented in random order). In the actual experiment 162 stimuli (the 3 syllables repeated 18 times each, 54 words, and 54 sentences) were presented in random order.

Stimuli were presented in 9 blocks, with 2 longer breaks after 3 blocks and shorter breaks (duration determined by the participant) in between the blocks. Three blocks lasted approximately 8 min, and it took approximately 30 min to complete the task, instructions and practice included.

The task was embedded in a child-friendly narrative to stimulate children’s attention and motivation to complete the task. Participants were told they are teaching 3 parrots how to “speak.” Their task was to wait for the parrot to start listening (when the cue appeared on the screen) and their instructions included keeping eye-contact with the parrot to make sure the bird is paying attention (to minimize movement-related artefacts in the recording). Furthermore, they were asked to repeat what they heard at a normal speaking loudness (i.e., to not mumble the syllables, words, or sentences) since the parrots will “not be able to learn if they don’t hear the speech properly” (to be able to record the production as clearly as possible). This also ensured the children were fully engaged in the task. Correct production was on average for children 88.41% and for adults 97.86%. At the end of each third block (i.e., before the longer breaks and the end of the test), the parrots “repeated” some of the heard sounds, which were new sounds created by raising the pitch of the original stimuli, to give the impression that the parrots were the ones repeating them. The first and second time it was the syllables, while at the end of the MEG recording it was one sentence from each syllable type.

Participants sat in a magnetically shielded, sound attenuated room under the MEG helmet, at a 68 degree position. The stimuli were presented through insert earphones (Rotel RA-1570 system; eartips were ER3-14B for children and ER3-14A for adults) at a comfortable loudness level. The participants sat 1 m from the projection screen. During measurement, a research assistant was also present in the room when necessary for the children. Presentation software (version 18.1; Neurobehavioral Systems, Inc., Albany, CA, USA) was used to present the stimuli, running on a Microsoft Windows computer (sound card: Sound Blaster Audigy RX; video card: NVIDIA Quadro K5200). Measurements were video monitored to make sure participants were paying attention and doing the task.

#### MEG recording

306-channel (102 magnetometers and 102 planar gradiometer pairs) MEG data were recorded in a magnetically shielded room using the Elekta Neuromag® TRIUX™ system (Elekta AB, Stockholm, Sweden) at the Centre for Interdisciplinary Brain Research, at the University of Jyväskylä, Finland.

The head position in relation to the sensors in the helmet was monitored continuously with five digitised head position indicator (HPI) coils attached to the scalp. Three HPI coils were placed on the forehead and one behind each ear. The position of the HPI coils was determined by three anatomic landmarks (nasion, left and right preauricular points) using the Polhemus Isotrak digital tracker system (Polhemus, Colchester, VT) at the beginning of the recording. An additional set of points (>100) randomly distributed over the scalp was also digitised. Electro-oculogram was recorded with two electrodes attached diagonally close to the left and right eyes and one ground electrode attached to the collar bone.

The sampling rate of the recording was 1000 Hz and a 0.03–330 Hz online band-pass filter was used.

### Data Analysis

#### Pre-processing

All data were pre-processed using the temporal extension of the signal space separation method with buffers of 30 s (Taulu & Kajola, 2005; Taulu et al., 2005) in Maxfilter 2.2™ (Elekta AB) to remove external interference and correct for head movements. Bad channels were identified and reconstructed by the Maxfilter program. Head position was estimated in 200 ms time-windows and 10 ms steps for movement compensation. Data were saved in three separate files containing three recording blocks. Initial head position of the first file was used for transforming the head position to the same position across the files.

Data were pre-processed using independent component analysis (ICA) using fastICA algorithm (Hyvärinen & Oja, 2000) to remove eye blinks, horizontal eye movements, and cardiac artifacts in MNE Python (0.16.2; Gramfort et al., 2013), and the separate MEG recordings were concatenated. The rest of the data analysis was done in the FieldTrip toolbox (Oostenveld et al., 2011) in MATLAB R2016 (https://www.mathworks.com/).

The continuous MEG recording was epoched to 100 ms before and 1,000 ms after the onset of sound in the syllable stimuli (for analysis of the evoked fields), and 100 ms before the onset of sound and 100 ms after the end of the sound in the word and sentence stimuli (for analysis of the frequency contents). Epochs were visually inspected and bad trials were rejected, with an average of 2.18% of epochs rejected for the children and 0.78% of epochs rejected for the adults. Data were low-pass filtered at 45 Hz. The epoched data was baseline corrected using the 100 ms preceding the onset of the stimuli.

We examined the data using two approaches. First, to examine how closely the brain follows the frequency contents of the speech signal, coherence was calculated between the MEG signal and the speech signal. Second, the evoked fields to the syllable stimuli were calculated to examine possible associations between the relatively well-known developmental changes of the evoked fields (particularly responses around 100 ms) and the coherence measures.

#### Coherence measures

We conducted coherence analysis at different frequency bands to investigate how brain activity changes while tracking the speech envelope of stimuli with different durations at different ages.

The speech stimuli were downsampled to 1000 Hz from 44.1 kHz. The absolute hilbert envelope was calculated for each stimulus separately in MATLAB (abs(hilbert(audiosignal))). The envelope was then appended to the epoched MEG data as a 307th channel.

Earlier studies looking into cross-correlations between the speech envelope and brain activity removed the first 250 ms of brain activity to avoid the onset evoked response (e.g., Abrams et al., 2008). However, the effects of the onset response on the coherence measures have not been reported before. Therefore, we performed the coherence analyses two times: first, for data without the evoked response, second, for the whole epoch length (see Supplementary Material 3). As shown in Supplementary Material 3, this did not have a large effect on the results. The results reported in the main text are based on analysis conducted using data where the evoked response was removed.

Frequency analysis of the data was done to compute the cross and power spectra of the trials using a multitaper frequency transformation method, where the maximum trial length was rounded up to the next power of 2 (cfg.pad = nextpow2) using FieldTrip’s ft_freqanalysis function, between 1 and 45 Hz with a 3 Hz smoothing and keeping the trials. This was followed by coherence analysis between the sound envelope and the MEG data using the ft_connectivityanalysis function.

Further, to see if the coherence between the brain and speech signals was significant at the individual level, we calculated 1,000 permutations of coherence, where the sound envelopes were randomly paired with the brain activity of another sound envelope, then compared with the original coherence value. For each participant at least one channel of the original speech–brain pair showed a coherence value larger than 95% of the permuted values (for visualization, see Supplementary Material 4).

To examine the effect of the stimulus length on the coherence values, we first checked the lengths of trials for word stimuli. Second, we cut out the end of the sentence stimuli to be of equal length with the word stimuli (i.e., the initial part of the sentence was used in the new analysis). We then recalculated the coherence between these shortened sentence stimuli and brain activity (see Supplementary Material 5). The results showed that shortened trials also had larger coherence values in both frequencies.

For further analyses, channels were grouped together by hemispheres (see Supplementary Material 6 for grouping of sensors across hemispheres). In the statistical analysis, data from magnetometers were averaged based on hemispheres and separated into two frequency bands: 1–3.5 Hz (delta), 4.5–8 Hz (theta).

For children, source reconstruction was based on their own T1 MRIs, while for adults the fsaverage brain template from Freesurfer (RRID: SCR_001847; Martinos Center for Biomedical Imaging, Charlestown, MA, USA) was used. Coregistration was done between the digitized head points and the brain template with 3-parameter scaling.

Source analysis was done using the ft_sourceanalysis, using the dynamic imaging of coherent sources method (Gross et al., 2001) between 1–8 Hz for every 0.5 Hz. The resulting coherence values were then averaged together according to the frequency band defined—delta band: 1–3.5 Hz, theta band: 4.5–8 Hz. The coherence values were then extracted based on the Desikan-Killiany Atlas (Desikan et al., 2006). Two regions of interest (ROIs) were selected a priori: the temporal area, including the superior temporal, transverse gyrus, and bank of superior temporal sulcus areas; and the inferior frontal area, including the pars opercularis, pars orbitalis, pars triangularis, and precentral areas (see, e.g., Molinaro et al., 2016).

#### Identification of responses around 100 ms to syllable stimuli and correlation with coherence values for the word and sentence stimuli

Trials for syllables were averaged together for each participant separately. Global mean field power (GMFP) was calculated for each group separately, and the time-window of auditory response was identified. Based on the GMFP peaks, the time-windows were defined by automatically finding the peak near 100 ms, and using a time-window of +/−25 ms for each hemisphere and group. Thus, the time-windows used in further analyses were 94–144 ms in the left hemisphere and 92–142 ms in the right hemisphere for adults, and 114–164 ms in the left and 113–163 ms in the right hemisphere for children. We averaged together the squared values from the temporal channels from the two hemispheres separately. The values were then correlated with the coherence values in the left and right hemispheres.

Topography of the averages was visually inspected to confirm the correct N1m response pattern or its equivalent in children. Earlier ERP/ERF research has shown that the N1m pattern reflects current direction towards inferior-posterior direction, and the opposite direction was referred to as P1m/P1m-like response. Indeed, averaging or grouping together opposite field patterns would obscure the outcome, and these patterns are likely to reflect distinct processes. Responses were separated based on hemisphere, then squared. The squared amplitude of the response was then correlated with the coherence values from the left and right hemispheres for the delta and theta bands.

A missing response could be due to noisy ERF signal. Therefore, signal-to-noise ratio was calculated by averaging and squaring together the baseline periods of the ERFs (time-window: −100–0 ms), and used as a covariate in separate ANOVAs to ensure that it was not the source of the differences found at sensor level. We found that it did not affect the significant effects.

Source analysis of the ERFs was done using ft_sourceanalysis, using the minimum-norm estimate (MNE) method (Hämäläinen & Ilmoniemi, 1994), and the power of each source component was calculated using ft_sourcedescriptives and used in the statistical analyses.

MNE source estimates were calculated for ERFs, and source power waveforms were extracted based on the Desikan-Killiany Atlas (Desikan et al., 2006). One ROI was selected a priori from the temporal areas around the auditory cortex including the temporal area, including the superior temporal, transverse gyrus, and bank of superior temporal sulcus areas, postcentral and supramarginal areas. The same time-windows were used as in the sensor level analysis. The literature clearly defines the sources of the N1m response near auditory cortex (Parviainen et al., 2019; Ponton et al., 2002). The ROIs for the coherence value analysis and ERFs were therefore expected to be slightly different with the former encompassing more frontal regions (Molinaro et al., 2016).

#### Statistical analyses

The age, hemisphere, and stimulus type effect on the coherence values for the different frequency bands were analysed in SPSS (IBM SPSS Statistics v. 24) using a 2 (Type: Word, Sentence) × 2 (Hemisphere: Left, Right) × 2 (Group: Children, Adults) repeated measures mixed ANOVA at both sensor and source levels. Significant interactions were further examined using independent samples *t* tests, and paired samples *t* tests where groups were involved in the interaction.

Pearson correlation was calculated between the coherence values at source level and the children’s ages in years rounded to months.

The averaged and squared responses around N1m to syllables were compared in a 2 (Hemisphere: Left, Right) × 2 (Group: Children, Adults) repeated measures mixed ANOVA. Further, Pearson correlation coefficients were calculated to examine the relationship between the peak amplitudes of the auditory responses around 100 ms and coherence values.

Pearson correlation coefficients were calculated to examine the relationship between the scores of three behavioural tests (RAN: objects subtests, NEPSY: Phonological processing and Sentence repetition subtests) and coherence values at source level.

Alpha level was 0.05. False discovery rate (FDR) correction for multiple comparisons was calculated for each analysis.

## RESULTS

### Coherence Between Brain and Speech Signals for Words and Sentences

#### Sensor level

**Figure 2.**
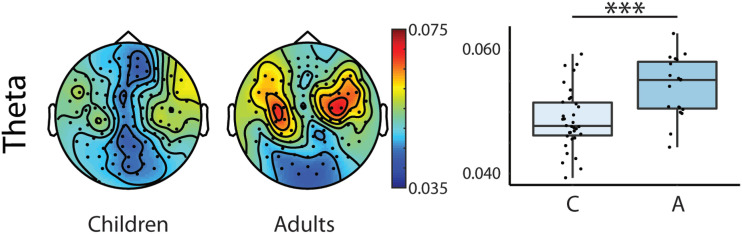
Topographic distribution of the coherence values and box plots of the theta frequency band showing Age main effect in the repeated measures mixed ANOVA for the two groups (Children, *N* = 34; Adults, *N* = 19) collapsed across hemispheres and stimulus types. Topographies: Warmer colours reflect higher coherence between the stimuli envelope and the brain data. Right box plots: Bold lines denote the median of the coherence values; the bottom and top edges of the box indicate the 25th and 75th percentiles, respectively. Light blue boxes show average coherence for children, dark blue boxes for adults (C = Children, A= Adults). (*** < 0.001)

**Figure 3.**
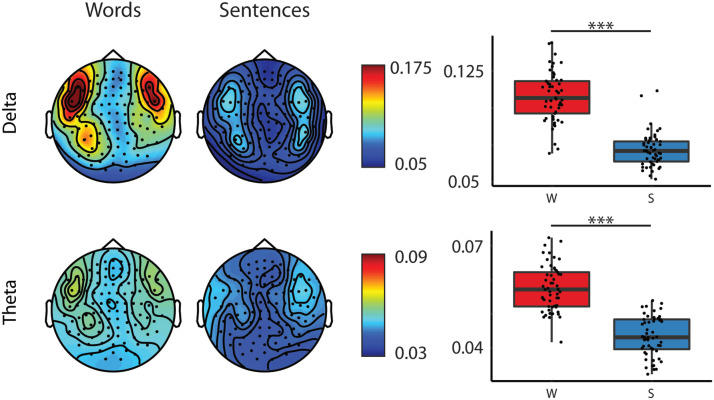
Topographic maps of coherence values of the two different frequency bands to word (W) and sentence (S) stimuli and box plots of averaged coherences for the delta and theta frequency bands collapsed across hemispheres and ages. Topographies: Warmer colours reflect higher coherence between the stimuli envelope and the brain data. Boxplots: Bold lines denote the median of the coherence values; the bottom and top edges of the box indicate the 25th and 75th percentiles, respectively. Red boxes represent average coherence values for words, and blue boxes for sentences. (*** < 0.001)

**Figure 4.**
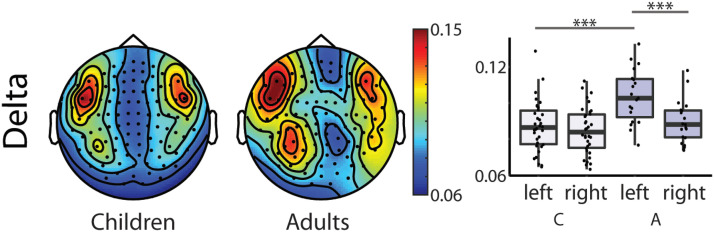
Topographic distribution of the coherence values and box plots of the delta frequency band showing a Hemisphere × Group interaction in the repeated measures mixed ANOVA (Children, *N* = 34; Adults, *N* = 19) collapsed across stimulus types. Topographies: Warmer colours reflect higher coherence between the stimuli envelope and the brain data. Right box plots: Bold lines denote the median of the coherence values; the bottom and top edges of the box indicate the 25th and 75th percentiles, respectively. Light purple boxes show average coherence for children, dark purple boxes for adults (C = Children, A = Adults). (*** < 0.001)

The results of the repeated measures ANOVA revealed first, that adults had the largest coherence values (see Tables 4 and 5, Group main effect; and Figure 2). Second, larger coherence values were observed for words as compared to sentences for both delta and theta frequency bands (see Tables 4 and 5, Type main effect; Figure 3). Further, we found that coherence values in the delta band were larger in the left compared to right hemispheres in adults’ brain responses and that adults had larger coherence values in the left hemisphere than children (see Table 4, Hemisphere × Group interaction; Figure 4).

**Table 4.** Results of repeated measures mixed ANOVA for the delta frequency band at sensor level

**Delta**	**Main effects and interactions**	***df***	***F* value**	***p* value**	**partial η^2^**
	***Type***	**1,51**	**227.754**	**0.000**	**0.817**
	***Hemisphere***	**1,51**	**11.631**	**0.001**	**0.186**
	***Group***	**1,51**	**12.739**	**0.001**	**0.200**
	*Type* × *Group*	1,51	0.295	0.589	0.006
	***Hemisphere*** × ***Group***	**1,51**	**5.822**	**0.019**	**0.102**
	*Type* × *Hemisphere*	1,51	3.670	0.061	0.067
	*Type* × *Hemisphere* × *Group*	1,51	0.996	0.323	0.019

*Note*. Bold values remained significant after false discovery rate correction.

**Table 5.** Results of repeated measures mixed ANOVA for the theta frequency band at sensor level

**Theta**	**Main effects and interactions**	***df***	***F* value**	***p* value**	**partial η^2^**
	***Type***	**1,51**	**259.307**	**0.000**	**0.836**
	*Hemisphere*	1,51	0.171	0.681	0.003
	***Group***	**1,51**	**14.089**	**0.000**	**0.216**
	*Type* × *Group*	1,51	0.836	0.365	0.016
	*Hemisphere* × *Group*	1,51	0.132	0.718	0.003
	*Type* × *Hemisphere*	1,51	0.017	0.896	0.000
	*Type* × *Hemisphere* × *Group*	1,51	0.051	0.822	0.001

*Note*. Bold values remained significant after false discovery rate correction.

Adults showed larger coherence values in the left hemisphere compared to the right hemisphere in the delta band (*t*(18) = 5.437, *p* = 0.000) when compared in a paired samples *t* test. Children’s coherence values did not differ significantly in the two hemispheres (*t*(33) = 0.730, *p* = 0.470). Further, independent samples *t* tests showed that adults had larger coherence values in the delta band in the left hemisphere compared to children (*t*(51) = −4.044, *p* = 0.000), and the groups did not differ significantly in their coherence values in the right hemisphere (*t*(51) = −1.386, *p* = 0.172).

#### Source level

**Figure 5.**
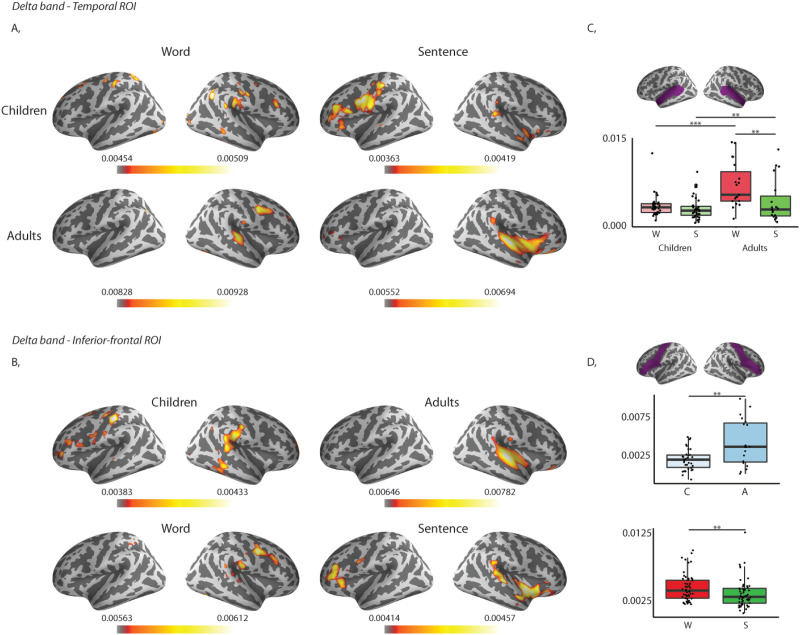
Left panels: Grand average of source level coherence values of children and adults to words and sentences. (A) In the delta frequency band in the temporal region of interest. (B) In the delta frequency band in the inferior-frontal region of interest; top row: grand averages of children and adults; bottom row: grand averages to words and sentences. Warmer colours reflect higher coherence between the stimuli envelope and the brain data. Right panels: Region of interest highlighted in purple (as defined in the Desikan-Killiany Atlas; Desikan et al., 2006). (C) Box plots of averaged coherence values in the delta frequency band in the temporal region collapsed across hemispheres. (D) Box plots of averaged coherence values in the delta frequency band in the inferior-frontal region collapsed across hemispheres and ages (top) or stimulus types (bottom). Bold lines denote the median of the coherence values; the bottom and top edges of the box indicate the 25th and 75th percentiles, respectively. W = words, S = sentences, C = children, A = adults. (*** < 0.001, ** < 0.01)

**Figure 6.**
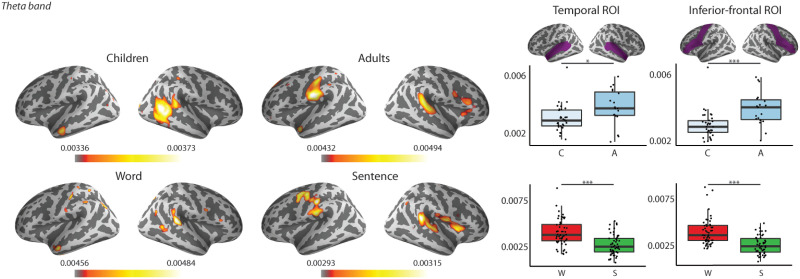
Left: Grand average of source level coherence values in the theta frequency band. Warmer colours reflect higher coherence between the stimuli envelope and the brain data. Top row: Grand averages of children and adults. Bottom row: Grand averages to words and sentences. Right: Region of interest highlighted in purple (as defined in the Desikan-Killiany Atlas; Desikan et al., 2006) and box plots of averaged coherences of the ROIs in the theta frequency band collapsed across hemispheres and ages (top) or stimulus types (bottom). Bold lines denote the median of the coherence values; the bottom and top edges of the box indicate the 25th and 75th percentiles, respectively. Top plot: Light blue boxes show average coherence for children (C), and dark blue boxes for the adults (A). Bottom plot: Red boxes represent average coherence values for words (W), and green boxes for sentences (S). (*** < 0.001, * < 0.05)

Similar to the sensor level, the results of the repeated measures ANOVA revealed that adults had the largest coherence values (see Tables 6 and 7, Group main effect; Figure 5) at source level. Second, larger coherence values were observed for words compared to sentences for both delta and theta frequency bands (see Tables 6 and 7: Type main effect; Figure 6). Third, we found that the adults had larger coherence values compared to children in the delta band in the temporal region in case of both words and sentences, and that adults had larger values for words than sentences (See Table 6, Type × Group interaction; Figure 5).

**Table 6.** Results of repeated measures mixed ANOVA for the delta frequency band at source level in the two regions of interests

**Delta** – **Temporal region**	**Main effects and interactions**	***df***	***F* value**	***p* value**	**partial η^2^**
	***Type***	**1,51**	**12.939**	**0.001**	**0.202**
	*Hemisphere*	1,51	5.266	0.026	0.094
	***Group***	**1,51**	**13.897**	**0.000**	**0.214**
	***Type*** × ***Group***	**1,51**	**6.519**	**0.014**	**0.113**
	*Hemisphere* × *Group*	1,51	1.727	0.195	0.033
	*Type* × *Hemisphere*	1,51	0.182	0.672	0.004
	*Type* × *Hemisphere* × *Group*	1,51	0.996	0.323	0.019

**Delta** – **Inferior-frontal region**	**Main effects and interactions**	***df***	***F* value**	***p* value**	**partial η^2^**
	***Type***	**1,51**	**9.143**	**0.004**	**0.152**
	*Hemisphere*	1,51	0.014	0.907	0.000
	***Group***	**1,51**	**13.476**	**0.001**	**0.209**
	*Type* × *Group*	1,51	1.291	0.261	0.025
	*Hemisphere* × *Group*	1,51	1.960	0.168	0.037
	*Type* × *Hemisphere*	1,51	0.737	0.395	0.014
	*Type* × *Hemisphere* × *Group*	1,51	0.037	0.849	0.001

*Note*. Bold values remained significant after false discovery rate correction.

**Table 7.** Results of repeated measures mixed ANOVA for the theta frequency band at source level in the two regions of interests

**Theta** – **Temporal region**	**Main effects and interactions**	***df***	***F* value**	***p* value**	**partial η^2^**
	***Type***	**1,51**	**44.799**	**0.000**	**0.468**
	*Hemisphere*	1,51	5.850	0.019	0.103
	***Group***	**1,51**	**6.849**	**0.012**	**0.118**
	*Type* × *Group*	1,51	2.131	0.151	0.040
	*Hemisphere* × *Group*	1,51	0.743	0.393	0.014
	*Type* × *Hemisphere*	1,51	0.253	0.617	0.005
	*Type* × *Hemisphere* × *Group*	1,51	0.190	0.665	0.004

**Theta** – **Inferior-frontal region**	**Main effects and interactions**	***df***	***F* value**	***p* value**	**partial η^2^**
	***Type***	**1,51**	**50.638**	**0.000**	**0.498**
	*Hemisphere*	1,51	0.540	0.465	0.011
	***Group***	**1,51**	**14.688**	**0.000**	**0.224**
	*Type* × *Group*	1,51	0.001	0.977	0.000
	*Hemisphere* × *Group*	1,51	0.865	0.357	0.017
	*Type* × *Hemisphere*	1,51	0.398	0.531	0.008
	*Type* × *Hemisphere* × *Group*	1,51	0.003	0.960	0.000

*Note*. Bold values remained significant after false discovery rate correction.

Post hoc independent samples *t* tests revealed that adults had significantly larger coherence values for words (*t*(51) = −4.467, *p* = 0.000) and for sentences (*t*(51) = −1.598, *p* = 0.002) compared to children. Paired samples *t* tests revealed that adults also had significantly larger coherence values for words compared to sentences (*t*(18) = 3.200, *p* = 0.005), and children’s coherence values did not differ significantly between words and sentences (*t*(33) = 1.000, *p* = 0.325).

Because the child group spanned a relatively large age range (4.7–9.3 years), we examined whether age was linearly related to changes in coherence values. We did not find any significant correlation between the observed coherence values and age (see Table 8).

**Table 8.** Results of correlations between the coherence values at source level and age in the children group

		**Correlation coefficient**	**Sig**	***N***
*Delta*	Temp	0.049	0.785	34
	Inf-front	−0.036	0.840	34
*Theta*	Temp	0.165	0.352	34
	Inf-front	0.056	0.752	34

*Note*. Sig = significance. Temp = Temporal. Inf-front = Inferior-frontal.

### Evoked Responses to Syllables

#### Sensor level

**Figure 7.**
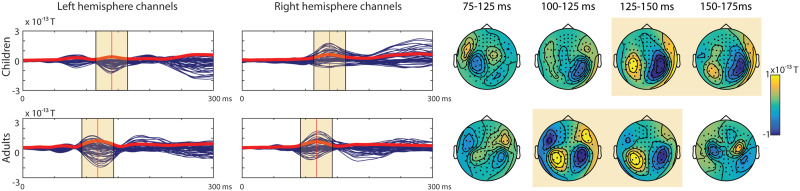
Blue butterfly plots of the group-averaged magnetometers with the global mean field power (GMFP) (red line) and topographic maps for the evoked responses to the syllable stimuli for the two age groups (Children, *N* = 34; Adults, *N* = 19). The yellow boxes highlight where the auditory response was expected in the groups based on the GMFP.

The averaged evoked responses’ topographies were typical of the N1m response in adults. In children the topography reminiscent of the N1m was slightly later in time in the right hemisphere. The left hemisphere showed a less clear pattern for children (see Figure 7). The topographies were also examined individually.

The averaged squared responses were compared in a 2 (Hemisphere: left, right) × 2 (Group: Children, Adults) repeated measures mixed ANOVA (see Table 9). No significant differences were found.

**Table 9.** Results of repeated measures mixed ANOVA for the averaged squared responses based on the GMFP peaks at sensor level

**Main effects and interactions**	***df***	***F* value**	***p* value**	**partial η^2^**
*Hemisphere*	1,51	0.013	0.531	0.000
*Group*	1,51	3.761	0.058	0.069
*Hemisphere* × *Group*	1,51	0.414	0.523	0.008

The averaged squared values were then correlated with the corresponding hemisphere’s coherence values in the frequency bands. No significant correlations were found. (For the table of correlation coefficients and *p* values, see Supplementary Material 7, Table 7.1.)

Topography of the averages was visually inspected then to confirm the correct N1m response pattern in each participant, for left and right hemispheres separately.

The N1m response in the left hemisphere was observed in 4 (11.76%) children, with an average latency of 130 ms, and 17 (89.47%) adults, with an average latency of 104 ms. Six (17.65%) children with an average latency of 151 ms showed an activation pattern with an opposite current direction to the adult-like N1m.

The N1m response in the right hemisphere was observed in 8 (23.53%) of the children’s evoked responses, with an average latency of 141 ms, and in 17 (89.47%) of the adults’ evoked responses, with an average latency of 105 ms. Five (14.71%) of the children with an average latency of 143 ms showed an activation pattern with an opposite current direction to the adult-like N1m.

**Figure 8.**
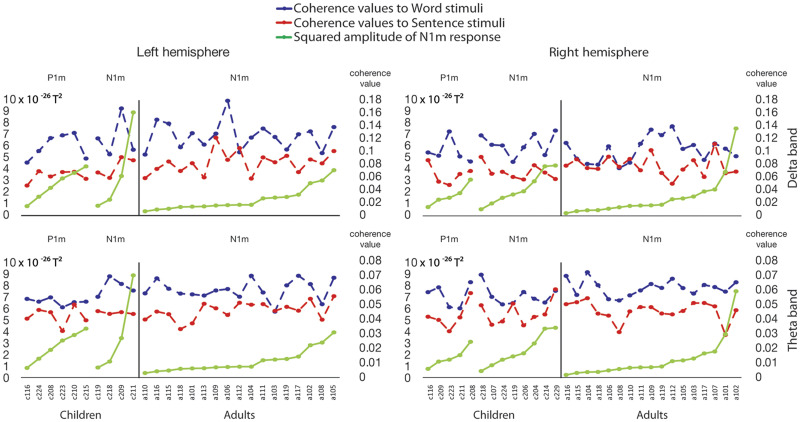
Line plots for coherence values in the left and right hemispheres to word and sentence stimuli in comparison to the squared amplitude of the N1m (T^2^) in the participants who showed a P1m or N1m pattern in their ERF responses. In each plot, the scale on the left side shows squared amplitude of N1m response, and the scale on the right side shows coherence values. Blue dashed line: coherence values to word stimuli; red dashed line: coherence values to sentence stimuli; green solid line: the squared N1m amplitude. Top row plots show coherence values for the delta band, left and right hemispheres respectively; bottom row plots show coherence values for the theta band, left and right hemispheres respectively. Values are organized in order of the squared amplitudes.

To examine whether the N1m amplitude and coherence values in the delta and theta bands would follow a similar developmental pattern, correlations were calculated to quantify the possible developmental relationship between the measures. Coherence values were plotted against the N1m responses in the child and adult groups (see Figure 8). No significant correlations were found between N1m amplitude to syllables and delta and theta coherence values to words and sentences in either the left or right hemispheres after correction for multiple comparisons. (For the table of correlation coefficients and *p* values, see Supplementary Material 7, Table 7.2.)

#### Source level

**Figure 9.**
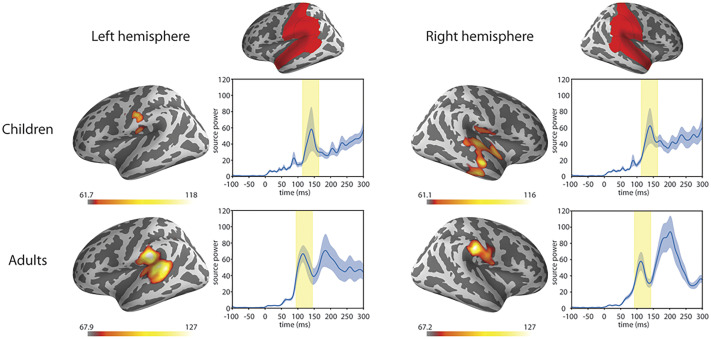
Grand averaged source level ERFs to syllables. Warmer colours reflect higher source power of the event-related field. The red areas highlighted were included in the region of interest (as defined in the Desikan-Killiany Atlas; Desikan et al., 2006). Right panels show the average source waveform (MNE estimate) extracted from the brain regions. The blue shading represents the standard error of the mean, and the yellow shading shows the time-windows used for the N1m response.

The responses were compared in a 2 (Hemisphere: left, right) × 2 (Group: Children, Adults) repeated measures mixed ANOVA (see Table 10 and Figure 9). No significant differences were found.

**Table 10.** Results of repeated measures mixed ANOVA for the averaged squared responses based on the GMFP peaks at source level

**Main effects and interactions**	***df***	***F* value**	***p* value**	**partial η^2^**
*Hemisphere*	1,51	0.3976	0.531	0.008
*Group*	1,51	0.105	0.747	0.002
*Hemisphere* × *Group*	1,51	3.469	0.068	0.064

The averaged power was then correlated with the corresponding hemisphere’s coherence values in the frequency bands. No significant correlations were found. (For the table of correlation coefficients and *p* values, see Supplementary Material 7, Table 7.3.)

### Correlations of Source Level Coherence with Behavioural Scores

**Figure 10.**
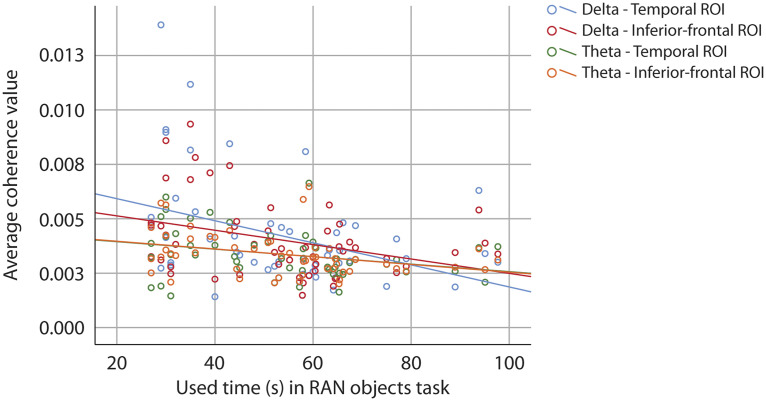
Scatter plot of correlation between performance on RAN objects and coherence values at source level. Blue dots and line represent coherence values from the temporal region of interest, and red dots and line represent values from the inferior-frontal region of interest in the delta frequency band. Green dots and line represent values from the temporal region of interest, and orange dots and line represent values from the inferior-frontal region of interest in the theta frequency band.

Behavioural scores in the Phonological processing and Sentence repetition tasks did not correlate with coherence values from the delta and theta bands. RAN objects did correlate inversely with both frequency bands and both ROIs (see Table 11 and Figure 10), but when age was controlled for, the correlation was no longer significant (see Table 12).

**Table 11.** Correlations between performance on RAN of objects (time in seconds) and the coherence values from the two regions of interest in the delta and theta frequency bands at source level

		**Correlation coefficient**	**Sig**	***N***
*Delta*	Temp	−**0.377**	**0.005**	**53**
	Inf-front	−**0.350**	**0.010**	**53**
*Theta*	Temp	−**0.292**	**0.034**	**53**
	Inf-front	−**0.330**	**0.016**	**53**

*Note*. Sig = significance. Temp = Temporal. Inf-front = Inferior-frontal.

**Table 12.** Correlations between performance on RAN of objects and the coherence values at source level controlled for age

		**Correlation coefficient**	**Sig**	***N***
*Delta*	Temp	−0.070	0.624	53
	Inf-front	0.020	0.888	53
*Theta*	Temp	−0.005	0.971	53
	Inf-front	0.055	0.699	53

*Note*. Sig = significance. Temp = Temporal. Inf-front = Inferior-frontal.

Coherence values were correlated with the child groups scores on NEPSY’s phonological processing task and sentence repetition task (see Tables 13 and 14). No significant correlations were found.

**Table 13.** Correlations between performance on the phonological processing task and the coherence values at source level

		**Correlation coefficient**	**Sig**	***N***
*Delta*	Temp	0.037	0.836	34
	Inf-front	−0.058	0.743	34
*Theta*	Temp	0.234	0.182	34
	Inf-front	0.131	0.462	34

*Note*. Sig = significance. Temp = Temporal. Inf-front = Inferior-frontal.

**Table 14.** Correlations between performance on the repetition of sentences task and the coherence values at source level

		**Correlation coefficient**	**Sig**	***N***
*Delta*	Temp	0.097	0.585	34
	Inf-front	0.200	0.256	34
*Theta*	Temp	0.076	0.670	34
	Inf-front	−0.055	0.757	34

*Note*. Sig = significance. Temp = Temporal. Inf-front = Inferior-frontal.

## DISCUSSION

This study investigated whether children and adults differ in overall brain activity as well as in left and right auditory cortex activity while listening to various speech units that are essential for spoken language processing. More specifically, we examined how auditory processing of words and sentences is reflected in the level of coherence and hemispheric lateralization across development, and how these are related to the processing of syllables, at both the sensor and source levels. To this end, two age groups were tested for comparison: children between the ages of 4.7 and 9.3 years and adults. Coherence is an interesting and useful measure of the brain’s ability to track the speech envelope across different frequency bands by quantifying the similarity in frequency content between brain activity and the speech envelope. The higher the coherence between brain activity and the speech envelope, the better the speech tracking.

First, we found an improvement with age in the brain’s ability to track speech evidenced as increased coherence values in the delta and theta frequency bands between brain signal and the speech envelope. Second, at the sensor level, where the whole hemispheres were examined, we found an interaction between hemispheres and age groups in the delta band with adults showing larger values at the left than the right hemisphere. However, at the source level, hemispheric differences in coherence values did not interact with age, which suggests no differences in maturation rates for the left and right auditory and frontal cortices in the degree to which the brain can synchronize to the speech envelope. Third, we also found differences in the coherence values observed for the word and sentence stimuli independent of age, although this was attributed to physical stimulus length rather than linguistic unit size, suggesting that the methodological approach should be taken into account when interpreting findings about speech perception. Last, we found no relationship between the general maturation of auditory processing and speech tracking as indicated by early ERFs to syllables.

Developmental differences were found as an overall increase in the coherence values in both the delta and theta frequency bands between adults and children, with adults showing largest values compared to children. Further, the topography of the coherence for these frequencies exhibited a clear pattern of auditory cortex activation at the sensor level. This was mostly confirmed by the source level analysis. The coherence values reflect how similar the frequency contents between the brain signal and the speech envelope are; therefore, our findings could be interpreted as increased precision of the auditory system to track the speech from childhood to adulthood. However, when examining the coherence values as a continuous variable within the child groups, we did not find any correlation between age and coherence values. There may be several reasons for the observed differences between adults and children.

First, basic auditory processing matures slowly with major changes in, for example, ERP responses noted at around ages 8–9 years with further changes until late adolescence (Ponton et al., 2000). This slow maturation of basic processing could affect the precision of speech processing in a bottom-up manner.

Second, the bottom-up process could be affected by genetically driven maturation or continued exposure to speech that refines the bottom-up pathway of the auditory system (Kuhl, 2000; Ponton et al., 2000). At the same time, continuous exposure to speech refines and changes the brain’s ability to perceive speech in a top-down manner as well (Kuhl, 2000). This environmental input would shape long-term memory representations, therefore affecting speech processing.

Last, the development of speech tracking may interact with other co-developing cognitive and language-related abilities (e.g., receptive and expressive vocabulary, speech motor and phonological developments) in addition to maturational factors such as age. There is for instance evidence that children initially process large units that are lexically based (e.g., words) before developing representations for smaller units (syllables, individual phonemes; for a review, see Vihman, 2017). This process may also be affected by reading acquisition that places emphasis on phonemes (e.g., Brennan et al., 2013; Popescu & Noiray, 2019; Ziegler & Goswami, 2005). Further, in speech production research investigating the size of coarticulatory units across age, Noiray and colleagues (Noiray et al., 2018; Noiray, Wieling et al., 2019) noted that children do not mature their coarticulatory patterns in a linear fashion. Instead, they found that preschoolers at the age of 3, 4, and 5 organised their speech in larger chunks compared to primary school children at the age of 7 and adults (Noiray et al., 2018; Noiray, Wieling et al., 2019).

In a subsequent study, Noiray and colleagues further demonstrated that the development of children’s phonological awareness, that is, the awareness that the native language, is composed of various size compounds (e.g., syllables, rhyme, and individual phonemes) and the ability to manipulate those units interacts with children’s speech motor organisation (Noiray, Popescu et al., 2019). Greater awareness of individual phonemes was associated with greater phonemic differentiation of articulatory gestures. To summarise, relationships between several cognitive and language-related abilities occur in the course of language acquisition, and they seem to evolve dynamically over time (e.g., Noiray, Popescu et al., 2019; Noiray, Wieling et al., 2019; Vihman, 2017). In future research, it will be important to investigate larger samples of children spanning kindergarten to primary school to better understand the dynamics of these relationships and how they contribute to the development of speech tracking specifically.

Our research provides supplementary information about the processing of various speech-sized units. More specifically, we confirmed the role of the lower frequency bands for sentence processing (Molinaro & Lizarazu, 2018; Ríos-López et al., 2020) and extended this finding to the processing of words. Indeed, there is evidence that theta and delta bands play a main role for parsing the continuous speech signal into linguistic and prosodic units (Poeppel, 2014; Poeppel & Assaneo, 2020). Thus, a developmental increase in brain coherence in these frequency bands could be associated with a development in the processing and awareness of those distinct speech units. While we did not find any significant correlation between children’s coherence values and their performance on phonological processing or sentence repetition, future studies should further examine the relationship among phonological awareness, reading, and speech tracking in the brain with larger samples of children and longitudinally.

We also found that coherence was higher for words than sentences for all frequency bands. This was somewhat surprising given longer stimuli should provide opportunities for brain activity to lock to the ongoing auditory signal. It is important, however, to note that after checking the coherence at the beginning of sentences trials (with the same length as used for words), we noted that coherence increased compared to the original values for sentences. This suggests that the higher coherence for words than sentences does not reflect differences that would be directly relevant for neural computation of linguistic units, but more the characteristics of calculating the coherence measure for short versus long stimuli. For example, longer stimuli provide greater chances for brain activity unrelated to stimulation to occur, with higher likelihood of this noise in the brain activity interacting with the coherence measure. Therefore, comparison of coherence measures across different length stimuli should be done with care, as pure physical length of the stimulus might have an effect on the results. In general, our findings confirm that speech tracking can happen at a shorter length, such as words, as well as at sentence level.

While we expected to find a significant interaction between the coherence values in the left and right hemispheres and the two age groups in the delta band, this difference was in the opposite direction from our predictions, where we expected larger values in the right hemisphere compared to the left, particularly in the adults (Luo & Poeppel, 2007). We observed significantly larger coherence values in the left hemisphere than right for adults in the delta band only at the sensor level—this was not observed at the source level. One possible reason for this difference between sensor and source level findings could be the selection of channels or regions used in the analysis. The sensor level comparison used an overall average of the hemispheres, while the source level focused on the temporal regions. At the source level, when focusing on the temporal regions, a hemispheric difference was found in the expected direction of larger right side activation compared to the left; however, the difference was no longer significant after FDR correction.

Finally, we investigated the overlap of different maturational processes across linguistic units by examining the age-related changes in brain activity around 125 ms (the time-window of the N1m response in adults to the syllables) and compared those to the coherence values for words and sentences. The amplitudes of evoked responses and the coherence values likely represent different neuronal mechanisms. The first presumably represents a more general maturation of the auditory and speech perception system, and the latter is likely linked to top-down processes such as comprehension of speech (Luo & Poeppel, 2007; Peelle et al., 2013; Ponton et al., 2000, 2002). We compared the development of the evoked responses and coherence using two approaches. In our first approach using GMFP, which included both the P1m and N1m responses, we found no significant difference between the groups. However, the P1m and N1m likely represent different computational processes of the sounds. In early childhood the auditory ERPs show prominent P1 and N2 peaks. During development, the P1 response shifts to earlier latencies accompanied by a decrease in amplitudes, and the prominent N1 response emerges to the waveform at around early school years (Albrecht et al., 2000; Ponton et al., 2000). Importantly, P1m of young children and N1m of older children and adults show very similar timing, obscuring the interpretation of purely GMFP-based interpretations (Parviainen et al., 2019).

Therefore, as a next step, we checked whether the spatial patterns and timings of responses in the left and right hemispheres for each individual matched with the expected N1m pattern based on the current direction in the magnetometer topography. We found no systematic correlation between the responses in the time-window of the first prominent evoked field (the N1m or P1m) and coherence between the speech envelope in the delta and theta bands. Although the correlations were not significant it should be noted that several factors might affect the result, such as sample size and methodology used. The ERFs and coherence were examined using different approaches (ERFs in the time domain and coherence in the frequency domain). It is possible that the use of these different approaches makes the measures difficult to compare directly. Taking this into consideration, our results suggest that the evoked response to syllables and the speech tracking might develop independently of each other and not share robust maturational mechanisms. If this is the case, the ERF amplitudes could reflect more bottom-up processes while the coherence values more top-down processes. Previous literature shows that ERFs are clearly modulated by the physical features of the sounds (Näätänen & Picton, 1986; Näätänen et al., 1997), and the speech envelope following seems to be linked to speech intelligibility and attention (Peelle et al., 2013).

Furthermore, especially in the case of younger children, while the GMFP did show a response around 100 ms, individual inspection of the responses showed that the response at the time was not actually an N1m response, but rather P1m. Taking this into account, it could perhaps explain why we found no differences between the groups when comparing the responses based on time-windows defined by only the peak in the GMFP.

Likewise, no hemispheric differences were observed in any of the age groups for the GMFP-based values of the evoked response, in contrast to a previous MEG study (Parviainen et al., 2019). However, the difference between these studies most likely reflects the chosen analysis approach. While the GMFP reflects the overall response strength at the sensor level, at the source level, measures of equivalent current dipoles depict the spatially specific amplitude values at different time points. Indeed, our data demonstrated a similarly delayed pattern of N1m topography in children, with more clear response in the right than left hemisphere, as was implied by Parviainen and colleagues (2011, 2019). Inspection of the responses themselves revealed that only about one third of the children actually had the N1m evoked response. Due to our sample size, comparison using brain responses of children who indeed produced the N1m response would be unbalanced, and future research should look into this comparison with a larger sample size for both groups.

One of the limitations of our study is related to the type of stimulus we used, as words uttered in isolation are more pronounced than those in a sentence. However, our post hoc comparison of the coherence values to sentences at word-length trials vs. sentence-length trials revealed that higher coherence was found at the beginning of the sentence regardless of stimulus type. It is possible the word level stimuli could be affected by their short length in the estimation of low frequencies, and therefore the results for the word stimuli should be regarded with caution. Another potential limitation is the number of participants limiting the power of the study to detect more subtle differences related to development or hemispheric processing of the different stimuli.

In summary, we investigated developmental differences in speech processing of various speech sized units that are linguistically relevant for spoken language processing, the syllable, word, and sentence, using MEG. We also examined how the hemispheric specialization is represented in brain responses and whether this specialization varies as a function of age. Overall, we found that both delta and theta frequencies show coherence with speech and seem to be important for speech processing. We also found developmental changes in the coherence values, which could reflect both bottom-up maturation and top-down refinement caused, for example, by continuous refinement of speech sound representations. Our data also suggest that the general functional maturation of the auditory cortices follows a different trajectory to that of the brain activity tracking the speech envelope.

## ACKNOWLEDGMENTS

The authors would like to thank Katja Koskialho, Sonja Tiri, Ainomaija Laitinen, Annamaria Vesterinen, Aino Sorsa, Maija Koskio, and Cherie Jenkins for their help with data collection. This work has been supported by the European Union projects Predictable (Marie Curie Innovative Training Networks, # 641858), and ChildBrain (Marie Curie Innovative Training Networks, #641652).

## FUNDING INFORMATION

Barbara Höhle, Horizon 2020 Framework Programme (https://dx.doi.org/10.13039/100010661), Award ID: 641858. Paavo Leppänen, Horizon 2020 Framework Programme (https://dx.doi.org/10.13039/100010661), Award ID: 641652.

## AUTHOR CONTRIBUTIONS

**Orsolya Beatrix Kolozsvári**: Conceptualization: Equal; Data curation: Equal; Investigation: Lead; Formal analysis: Equal; Methodology: Equal; Project administration: Equal; Software: Equal; Visualisation: Lead; Writing–Original Draft: Equal; Writing–Review & Editing: Equal. **Weiyong Xu**: Conceptualization: Equal; Data curation: Equal; Formal analysis: Equal; Methodology: Equal; Software: Equal; Visualisation: Supporting; Writing–Original Draft: Equal; Writing–Review & Editing: Equal. **Georgia Gerike**: Formal analysis: Equal; Methodology: Equal; Visualisation: Supporting; Writing–Review & Editing: Equal. **Tiina Parviainen**: Conceptualization: Equal; Methodology: Supporting; Supervision: Supporting; Writing–Review & Editing: Equal. **Lea Nieminen**: Conceptualization: Equal; Methodology: Supporting; Writing–Review & Editing: Equal. **Aude Noiray**: Supervision: Supporting; Writing–Review & Editing: Equal. **Jarmo Hämäläinen:** Conceptualization: Equal; Formal analysis: Equal; Funding acquisition: Lead; Methodology: Equal; Project administration: Equal; Supervision: Lead; Writing–Original Draft: Equal; Writing–Review & Editing: Equal.

## TECHNICAL TERMS

Functional near-infrared spectroscopy (fNIRS): Functional neuroimaging technique using near-infrared spectroscopy where cerebral hemodynamic responses are measured.
Coherence: A value that reflects how similar the oscillatory activity present in two signals is.
Event-related potential (ERP): The brain response to a presented stimulus, measured using electroencephalography (EEG).
N1m: Auditory evoked response related to N1 response, measured using magnetoencephalography
Global mean field power (GMFP): A measure used to characterize global MEG activity.
