# Supplementary material for: Coherence Between Brain Activation and Speech Envelope at Word and Sentence Levels Showed Age-Related Differences in Low Frequency Bands
Source: Neurobiol Lang (Camb). 2021 May 7;2(2):226–53. doi: 10.1162/nol_a_00033 (PMC10158622; doi:10.1162/nol_a_00033)
Supplement: Supplementary file 1 [file nol-2-2-226-s001.pdf]

SUPPLEMENTARY MATERIAL 1. DESCRIPTION OF BEHAVIOURAL TESTS

BLOCK DESIGN

In the block design task, participants were instructed to arrange red and white blocks to form a design and the task consists of building the same patterns. In more advanced sections the participants were only shown the design in a figure and they had to build it. Cut-off point was 3 items assembled incorrectly.

VOCABULARY

In the expressive vocabulary test participants heard a word and they had to describe the meaning of that word. Cut-off point for the task was five items in a row incorrectly described

DIGIT SPAN

Forward and backward digit span subtests (for children) as well as sequencing subtask (for adults) were used. Participants were instructed to repeat a series of digits uttered by the experimenter in the same or reversed or ascending order. The number of items to be repeated ranged from two to nine in the task, with 2 sequences for each length of series. Incorrect repetition of both sequences in a set was the cut-off point for this task.

NEPSY: REPETITION OF NONSENSE WORDS

This subtest was used to assess phonological encoding and decoding. Participants were asked to repeat pseudo- or non-words presented out loud. Number of maximum items was 16.

NEPSY: OROMOTOR TASK

This subtest is designed to assess oromotor coordination. Participants were asked to repeat articulatory sequences until the required number of repetitions is reached. Cut-off point for this task was 4 incorrect items in a row.

NEPSY-II: VISUO-MOTOR TASK - CAR DESIGN, MOTORCYCLE DESIGN

This timed subtest is designed to assess graphomotor speed and accuracy. The participant used his or her preferred hand to draw lines inside of tracks as quickly as possible. Two types of tests were used: car and motorcycle.

NEPSY-II: PHONOLOGICAL PROCESSING

Two subtests were used. In Word Segment Recognition, participants were asked to identify words from word segments. In Phonological Segmentation the participants are asked to repeat a word and then to create a new word by omitting a syllable or a phoneme or by replacing one phoneme in a word another. Cut-off point for these tasks was 6 incorrect responses in a row.

NEPSY-II: REPETITION OF SENTENCES

Participants listened to increasingly long sentences and had to try to repeat them as accurately as possible. Cut-off point for this task was 4 incorrect responses in a row.

RAN: OBJECTS

Rapid automatized naming (RAN) task (pictures: ball, car, fish, house, pencil) was used with a 10×5-item matrix (Denckla and Rudel, 1976). Total matrix completion time (in seconds) was used as a measure.

RAN: LETTERS

Rapid automatized naming (RAN) task (letters: A, O, P, S, T) was used with a 10×5-item matrix (Denckla and Rudel, 1976). Total matrix completion time (in seconds) was used as a measure.

LETTER KNOWLEDGE TASK

Letter naming was measured by presenting 29 capital letters one by one. The sum of correct answers (the use of a phoneme and the use of a letter name would both be coded as correct responses but all of the responses were given using letter names) was used as a measure.

LUKILASSE: WORD READING

Participants were asked to read a list of words, using a standardized test of word list reading (Lukilasse; Häyrinen, Serenius-Sirve, & Korkman, 1999). Percentile based on number of correctly read words in 45s is reported.

LUKILASSE: DICTATION

Participants were asked to write words and pseudowords (number of syllables from one up to three) from dictation.

PSEUDOWORD LIST READING

Participants were asked to read a list of pseudowords, based on Tests of Word Reading Efficiency (TOWRE; Torgesen et al., 1999). Number of correctly read non-words in 45 s was used as the score.

PSEUDOWORD TEXT READING

Participants were asked to read a text consisting of pseudowords (Eklund, Torppa, Aro, Leppänen, & Lyytinen, 2015) and number of correctly read words was used as the score.
