# Supplementary material for: Coherence Between Brain Activation and Speech Envelope at Word and Sentence Levels Showed Age-Related Differences in Low Frequency Bands
Source: Neurobiol Lang (Camb). 2021 May 7;2(2):226–53. doi: 10.1162/nol_a_00033 (PMC10158622; doi:10.1162/nol_a_00033)
Supplement: Supplementary file 2 [file nol-2-2-226-s002.pdf]

Supplementary Material 2. Table of stimuli used in the experiment

Supplementary Table 2.1 All stimuli used in the experiment and their English translation

		Syllables			
**ka**		**pa**		**ta**	
		**Words & *English translation***			
**kala**	*fish*	**paju**	*willow*	**talo**	*house*
**kana**	*chicken*	**pala**	*piece*	**tarina**	*story*
**kani**	*rabbit*	**pata**	*casserole, pot*	**tapetti**	*wallpaper*
**katu**	*street*	**pahis**	*bad guy*	**taide**	*art*
**kameli**	*camel*	**paperi**	*paper*	**taika**	*magic, spell*
**kaneli**	*cinnamon*	**paketti**	*parcel*	**taulu**	*painting, picture, blackboard*
**kaali**	*cabbage*	**palikka**	*brick*	**taivas**	*sky; heaven*
**kauha**	*ladle, scoop*	**paita**	*shirt*	**taikina**	*pastry, dough*
**kauppa**	*shop, store*	**paisti**	*roast*	**taikuri**	*magician*
**kakku**	*cake*	**pallo**	*ball*	**tahna**	*paste*
**kahvi**	*coffee*	**palmu**	*palm tree*	**tahra**	*stain*
**kannu**	*jug*	**pannu**	*frying pan, pot*	**takka**	*fireplace*
**karhu**	*bear*	**pappa**	*grandfather*	**takki**	*coat*
**kassi**	*bag*	**parta**	*beard*	**talvi**	*winter*
**kasvi**	*plant*	**patja**	*mattress*	**tammi**	*oak*
**katti**	*cat*	**patsas**	*statue*	**tarra**	*sticker*
**karkki**	*sweet, candy*	**pannari**	*oven pancake*	**tassu**	*paw*
**kattila**	*pot, pan*	**pantteri**	*panther*	**tatti**	*boletus*

Sentences & ***English translation***

**Kala on akvaariossa.**	*The fish is in the aquarium.*	**Paju on taipuisa puu.**	*A willow is a flexible tree.*	**Talo on aivan uusi.**	*The house is a brand new one.*
**Kana on kotieläin.**	*Chicken is a domestic animal.*	**Pala on kovin pieni.**	*The piece is very small.*	**Tarina on jännittävä.**	*The story is exciting.*
**Kani on pitkäkorvainen.**	*A rabbit has long ears.*	**Pata on liedellä.**	*The pot is on the stove.*	**Tapetti on raidallinen.**	*The wallpaper is stripy.*
**Katu on melkein tyhjä.**	*The street is almost empty.*	**Pahis on rikollinen.**	*A bad guy is a criminal.*	**Taide on kallista.**	*Art is expensive.*
**Kameli on ruskea eläin.**	*A camel is a brown animal.*	**Paperi on valkoista.**	*Paper is white.*	**Taika on ihmeellinen**	*Magic is wonderful.*
**Kaneli on mauste.**	*Cinnamon is a spice.*	**Paketti on painava.**	*The parcel is heavy.*	**Taulu on hyvin värikäs.**	*The board is colourful.*
**Kaali on vihannes.**	*Cabbage is a vegetable.*	**Palikka on punainen.**	*The brick / building block is red.*	**Taivas on pilvinen.**	*The sky is cloudy.*
**Kauha on puinen.**	*The scoop is made of wood.*	**Paita on kirjava.**	*The shirt is multi-coloured.*	**Taikina on hyvää.**	*The pastry is delicious.*
**Kauppa on kiinni.**	*The shop is closed.*	**Paisti on uunissa**	*The roast is in the oven.*	**Taikuri on salaperäinen.**	*The magician is mysterious.*
**Kakussa on kauniit koristeet.**	*The cake is beautifully garnished.*	**Pallo on korissa.**	*The ball is in the basket*	**Tahna on tahmeaa.**	*The paste is sticky.*
**Kahvi on kuumaa.**	*The coffee is hot.*	**Palmu on puu.**	*A palm tree is a tree.*	**Tahra on vaikea irrottaa.**	*The stain is hard to remove.*
**Kannussa on mehua.**	*There is juice in the jug.*	**Pannu on kuuma.**	*The frying pan is hot.*	**Takka on lämmin.**	*The fireplace is warm.*
**Karhu on metsän kuningas.**	*The bear is the king of the woods.*	**Pappa on jo vanha.**	*Grandfather is already old.*	**Takki on liian pieni.**	*The coat is too small.*
**Kassi on ihan tyhjä.**	*The bag is empty.*	**Parta on tosi pitkä.**	*The beard is very long.*	**Talvi on kylmä vuodenaika.**	*Winter is a cold season.*
**Kasvi on vihrea.**	*The plant is green.*	**Patja on epämukava.**	*The mattress is uncomfortable.*	**Tammi on todella vanha.**	*The oak is really old.*
**Katti on kissa.**	*A cat is a cat.*	**Patsas on keskellä toria.**	*The statue is in the middle of the square.*	**Tarra on kiiltävä.**	*The sticker is glossy.*
**Karkki on makea.**	*The candy is sweet.*	**Pannari on herkullista.**	*Oven pancake is delicious.*	**Tassu on kipeä.**	*The paw hurts.*
**Kattila on tehty kuparista.**	*The pot is coated with copper.*	**Pantteri on ketterä eläin**	*A panther is an agile animal.*	**Tatti on sieni**	*Boletus is one kind of a mushroom.*
