# Supplementary material for: Coherence Between Brain Activation and Speech Envelope at Word and Sentence Levels Showed Age-Related Differences in Low Frequency Bands
Source: Neurobiol Lang (Camb). 2021 May 7;2(2):226–53. doi: 10.1162/nol_a_00033 (PMC10158622; doi:10.1162/nol_a_00033)
Supplement: Supplementary file 3 [file nol-2-2-226-s003.pdf]

SUPPLEMENTARY MATERIAL 3. RESULTS OF ANALYSIS WHERE EVOKED RESPONSES WERE KEPT

COHERENCE VALUES

Repeated measures mixed ANOVA (2 (type) x 2 (hemisphere) x 3 (age)) was conducted to investigate the differences in development for processing of stimulus content and hemispheres. False discovery rate (FDR) was calculated for correction for multiple comparisons.

Adults had the largest coherence values on average, while pre-school age group had the lowest values out of the three age groups in the delta and theta bands (see Supplementary Table 3.1: Age main effect; and Supplementary Figure 3.1).

Our results showed larger coherence values for words than for sentences for all frequency bands (Supplementary Table 3.1: Type main effect; Supplementary Figure 3.2).

Supplementary Table 3.1. Results of repeated measures mixed ANOVA for each frequency band.

Main effects		*df*	*F-value*	*p-value*	*partial η^2*
Hemisphere	*Delta (1-4 Hz)*	1,50	5.416	0.024	0.098
	Theta (4-8 Hz)	1,50	0.001	0.982	0.000
	Alpha (8-12 Hz)	1,50	0.741	0.394	0.015
	Low Beta (12-20 Hz)	1,50	0.001	0.971	0.000
	High Beta (20-30 Hz)	1,50	1.835	0.182	0.035
	Low Gamma (30-45 Hz)	1,50	0.039	0.844	0.001
Age	***Delta (1-4 Hz)***	**2,50**	**9.671**	**<0.001**	**0.279**
	Theta (4-8 Hz)	**2,50**	**5.065**	**0.010**	**0.168**
	Alpha (8-12 Hz)	2,50	0.071	0.931	0.003
	Low Beta (12-20 Hz)	2,50	0.130	0.878	0.005
	High Beta (20-30 Hz)	2,50	1.908	0.159	0.071
	Low Gamma (30-45 Hz)	2,50	0.058	0.944	0.002
Type	***Delta (1-4 Hz)***	**1,50**	**396.743**	**<0.001**	**0.888**
	Theta (4-8 Hz)	**1,50**	**556.609**	**<0.001**	**0.918**
	Alpha (8-12 Hz)	**1,50**	**211.793**	**<0.001**	**0.809**
	Low Beta (12-20 Hz)	**1,50**	**563.857**	**<0.001**	**0.919**
	High Beta (20-30 Hz)	**1,50**	**381.404**	**<0.001**	**0.884**
	Low Gamma (30-45 Hz)	**1,50**	**731.594**	**<0.001**	**0.936**
Interactions					
Hemisphere x Age	*Delta (1-4 Hz)*	2,50	3.399	0.041	0.120
	Theta (4-8 Hz)	2,50	0.063	0.939	0.002
	Alpha (8-12 Hz)	2,50	0.116	0.890	0.005
	Low Beta (12-20 Hz)	2,50	0.856	0.431	0.033
	High Beta (20-30 Hz)	2,50	2.136	0.129	0.079
	Low Gamma (30-45 Hz)	2,50	1.963	0.151	0.073
Hemisphere x Type	*Delta (1-4 Hz)*	1,50	1.398	0.243	0.027
	Theta (4-8 Hz)	1,50	3.147	0.082	0.059
	Alpha (8-12 Hz)	1,50	0.432	0.514	0.009
	Low Beta (12-20 Hz)	1,50	2.285	0.137	0.044
	High Beta (20-30 Hz)	1,50	0.227	0.636	0.005
	Low Gamma (30-45 Hz)	1,50	0.457	0.502	0.009
Age x Type	*Delta (1-4 Hz)*	2,50	3.709	0.031	0.129
	Theta (4-8 Hz)	2,50	0.100	0.905	0.004
	Alpha (8-12 Hz)	2,50	0.918	0.406	0.035
	Low Beta (12-20 Hz)	2,50	1.233	0.300	0.047

	High Beta (20-30 Hz)	2,50	0.058	0.943	0.002
	Low Gamma (30-45 Hz)	2,50	3.270	0.046	0.116
Hemisphere x Age x Type	*Delta (1-4 Hz)*	2,50	0.882	0.420	0.034
	Theta (4-8 Hz)	2,50	1.623	0.208	0.061
	Alpha (8-12 Hz)	2,50	0.598	0.554	0.023
	Low Beta (12-20 Hz)	2,50	0.840	0.438	0.033
	High Beta (20-30 Hz)	2,50	0.086	0.918	0.003
	Low Gamma (30-45 Hz)	2,50	0.516	0.600	0.020

Note. Bold values remained significant after false discovery rate correction.

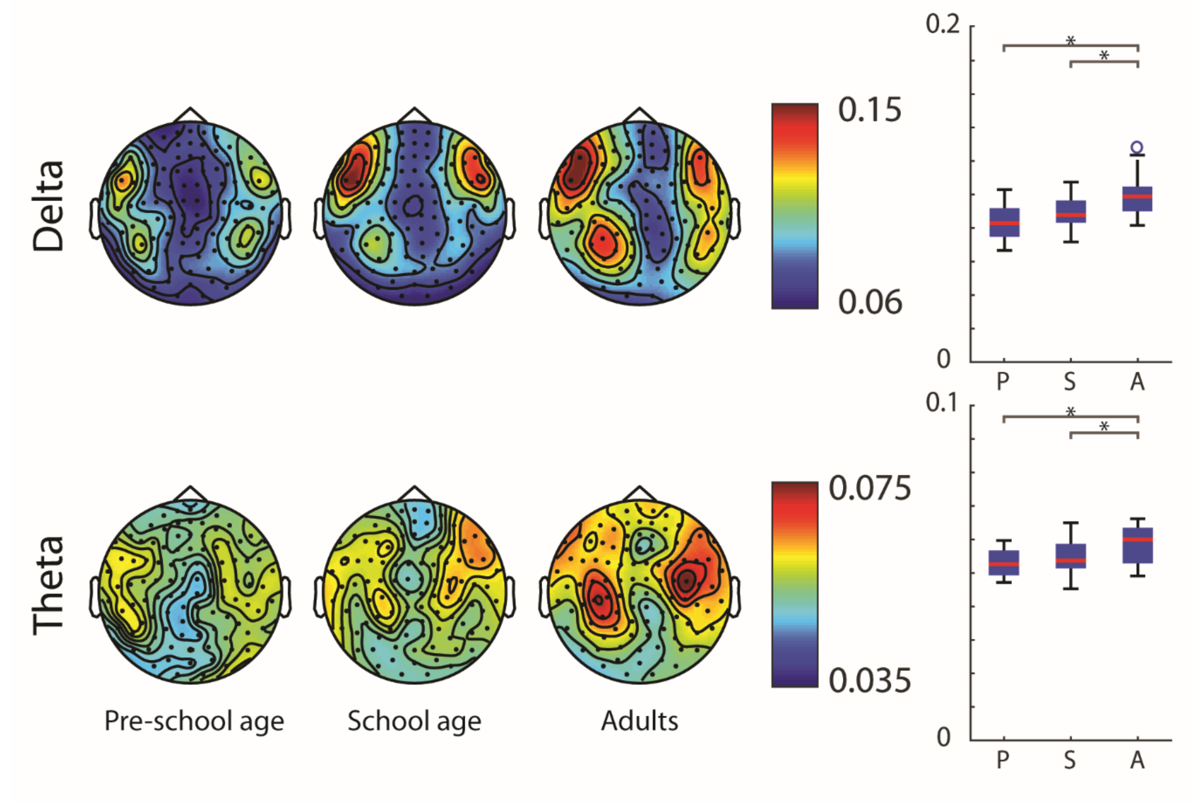

Supplementary Figure 3.1 Topographic distribution of the coherence values and box plots of the delta and the theta frequency bands showing Age main effect in the repeated measures mixed ANOVA for the three age groups (Pre-school age, N=12; School age, N=22; Adults, N=19) collapsed across hemispheres and stimulus types. Topographies: warmer colours reflect higher coherence between the stimuli envelope and the brain data. Right box plots: Red bolded lines denote the median of the coherence values, the bottom and top edges of the box indicate the 25th and 75th percentiles, respectively. (P = Pre-school age, S= School age, A= Adults).

The main effect of Age found in the ANOVA was further examined with Independent samples T-tests.

Adults had larger coherence values than pre-school age children in both the delta ($t(29) = -3.083$, $p = 0.004$) and theta bands ($t(29) = -3.887$, $p = 0.001$), Similarly, coherence values were larger for adults than for school age children in both the delta ($t(39) = -3.192$, $p = 0.003$) and theta bands ($t(39) = -2.229$, $p = 0.032$). No significant differences were found between the other age comparison (delta: pre-school age group vs school age group ($t(32) = -1.556$, $p = 0.130$); theta: pre-school age group vs school age group ($t(32) = -1.079$, $p = 0.288$)).

Overall, adults had the largest coherence values on average, while pre-school age children had the lowest average values out of the three age groups.

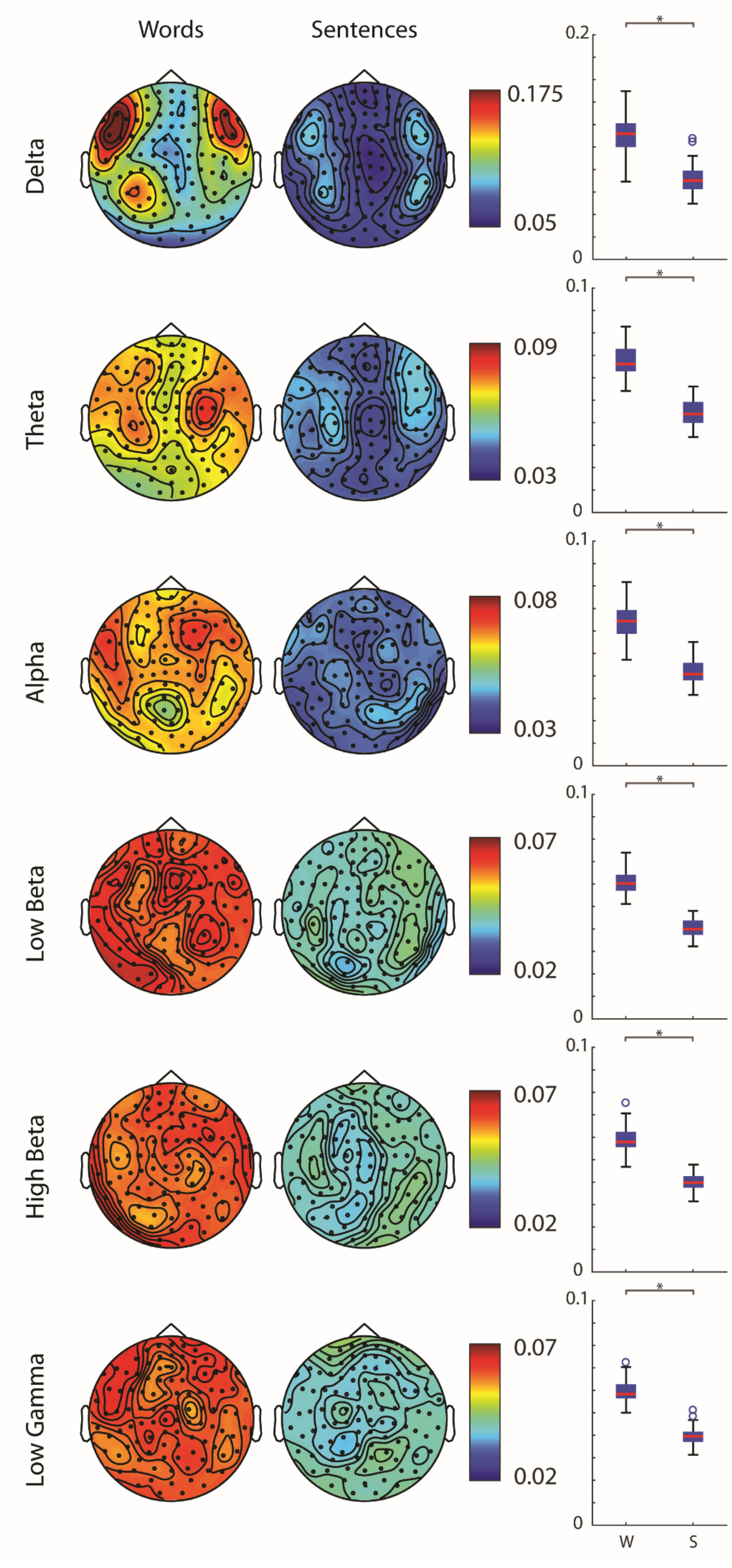

Words
Sentences
Delta
0.175
0.05
Theta
0.09
0.03
Alpha
0.08
0.03
Low Beta
0.07
0.02
High Beta
0.07
0.02
Low Gamma
0.07
0.02
0.2
0.1
0
*
W
S

Supplementary Figure 3.2 Topographic distribution of the coherence values and box plots of all frequency bands showing Type main effect in the repeated measures mixed ANOVA collapsed across hemispheres and ages. Topographies: warmer colours reflect higher coherence between the stimuli envelope and the brain data. Boxplots: Red bolded lines denote the median of the coherence values, the bottom and top edges of the box indicate the 25th and 75th percentiles, respectively. (W: words, S: sentences)
