# Supplementary figures and images for: Coherence Between Brain Activation and Speech Envelope at Word and Sentence Levels Showed Age-Related Differences in Low Frequency Bands
Source: Neurobiol Lang (Camb). 2021 May 7;2(2):226–53. doi: 10.1162/nol_a_00033 (PMC10158622; doi:10.1162/nol_a_00033)

SUPPLEMENTARY MATERIAL 4. RESULTS OF PERMUTATION

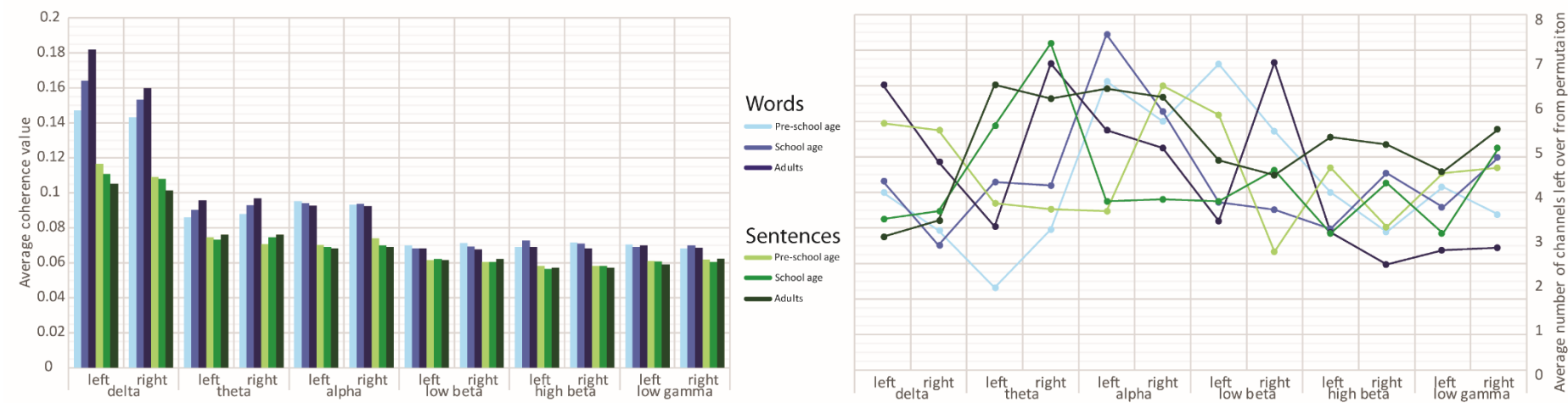

Supplement: Supplementary file 4 [file nol-2-2-226-s004.pdf]
