# Supplementary material for: Coherence Between Brain Activation and Speech Envelope at Word and Sentence Levels Showed Age-Related Differences in Low Frequency Bands
Source: Neurobiol Lang (Camb). 2021 May 7;2(2):226–53. doi: 10.1162/nol_a_00033 (PMC10158622; doi:10.1162/nol_a_00033)
Supplement: Supplementary file 5 [file nol-2-2-226-s005.pdf]

SUPPLEMENTARY MATERIAL 5. EFFECT OF STIMULUS LENGTH ON THE COHERENCE VALUES

To examine the effect of the stimulus length on the coherence values we first checked the lengths of trials for word stimuli. Second, we cut out the end of the sentence stimuli to be of equal length with the word stimuli (i.e., the initial part of the sentence was used in the new analysis). We then recalculated the coherence between these shortened stimuli and the brain activity (See Supplementary Figure 5.1).

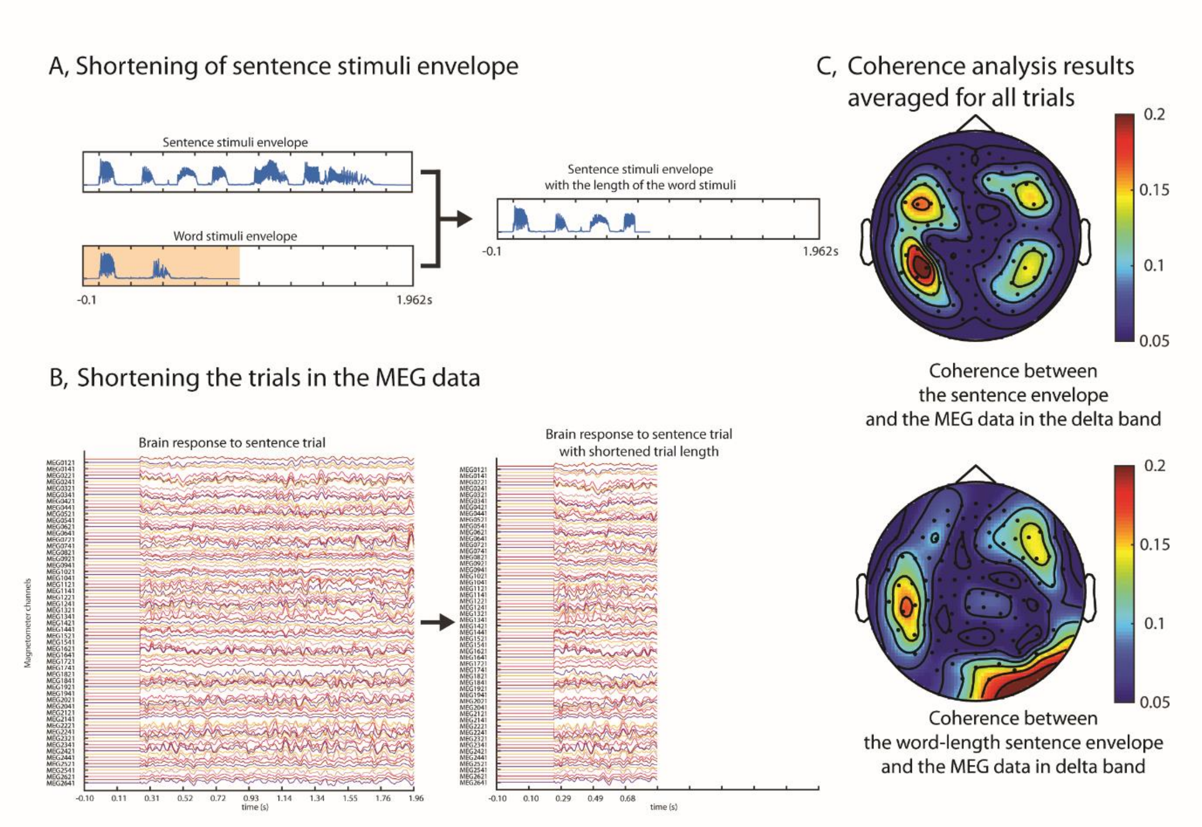

Supplementary Figure 5.1 A schematic representation of how the sentence stimuli trials were shortened to the length of the word stimuli trials and used in the coherence calculations using an example data. A, The length of the word stimuli (which is the same as the first word of the sentence stimuli) is used to determine the shortened length to be used for the word-length sentence stimuli envelope. B, The trials of the brain response are shortened to the same length C, Coherence is calculated for the shortened sentence stimuli envelopes and are then compared with the coherence values of the original length sentence stimuli.

The coherence values were then compared with the original sentence duration values. It could be that having a longer stimulus gives rise to more time points where the brain signal can vary independently from the speech signal. This would lead to lower coherence values for the sentence level stimuli. To examine this, paired t-tests were calculated between the shortened

and original sentence coherence values collapsed across age and hemisphere. The shortened sentences had significantly larger coherence values in both delta and theta bands (See Supplementary Table 5.1, and Supplementary Figure 5.2).

Supplementary Table 5.1 Results of paired samples T-tests comparing the coherence values to the shortened (word length) and the original sentence stimuli collapsed across hemispheres.

	df	*t-value*	*p-value*
Delta (1-4 Hz)	**52**	**-3.274**	**0.002**
Theta (4-8 Hz)	**52**	**-19.798**	**0.000**

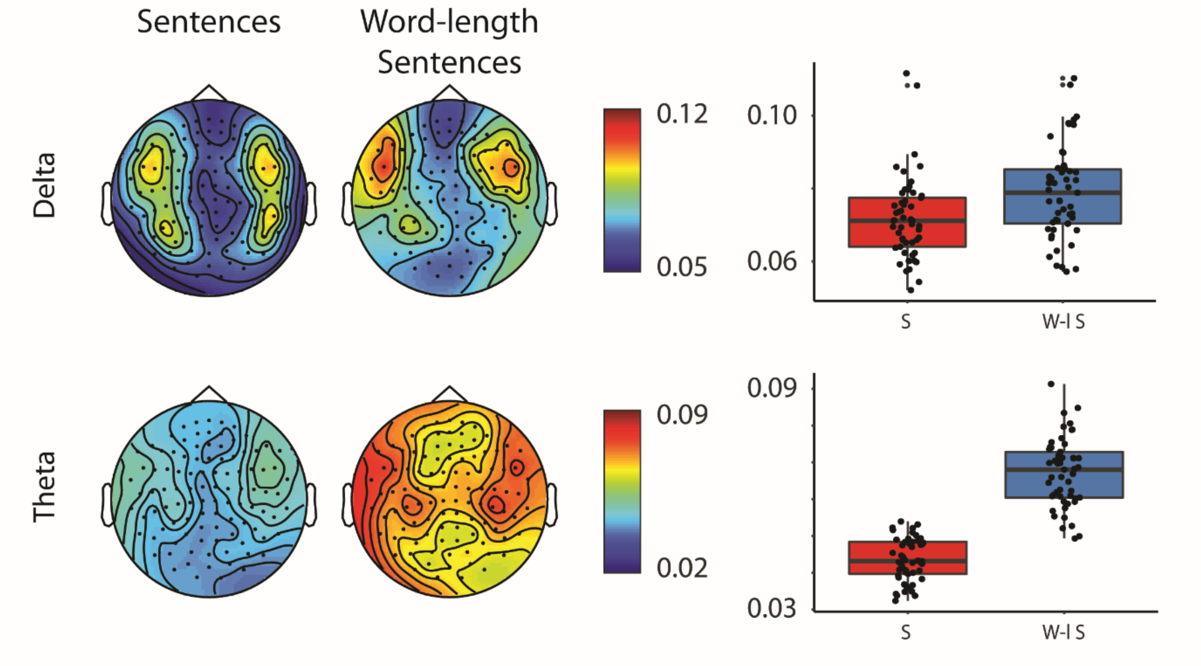

Supplementary Figure 5.2 Topographic maps of coherence values of the two frequency bands to sentence (S) stimuli and the shortened (word-length) trials for sentence (W-l S) stimuli and box plots of averaged coherences for the frequencies collapsed across hemispheres and ages. Topographies: warmer colours reflect higher coherence between the stimuli envelope and the brain data. Boxplots: Boldlines denote the median of the coherence values, the bottom and top edges of the box indicate the 25th and 75th percentiles, respectively. Red boxes represent average coherence values for sentences, green boxes for sentences with shortened trial lengths.
