# Supplementary material for: Coherence Between Brain Activation and Speech Envelope at Word and Sentence Levels Showed Age-Related Differences in Low Frequency Bands
Source: Neurobiol Lang (Camb). 2021 May 7;2(2):226–53. doi: 10.1162/nol_a_00033 (PMC10158622; doi:10.1162/nol_a_00033)
Supplement: Supplementary file 6 [file nol-2-2-226-s006.pdf]

SUPPLEMENTARY MATERIAL 6 MEG SENSOR LAYOUT

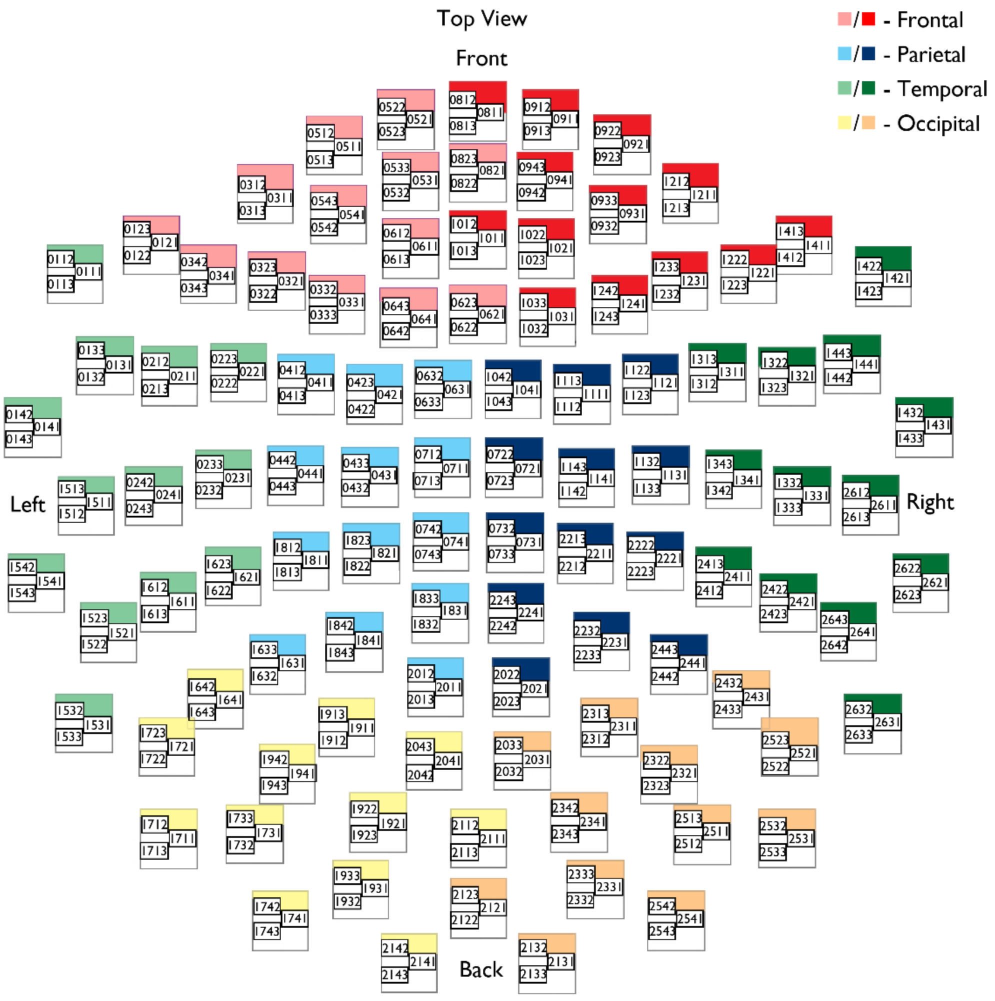

Supplementary Figure 6.1 MEG sensor layout: division to the left and right sensor space. Channel numbers are marked for each location.
