# Supplementary material for: Coherence Between Brain Activation and Speech Envelope at Word and Sentence Levels Showed Age-Related Differences in Low Frequency Bands
Source: Neurobiol Lang (Camb). 2021 May 7;2(2):226–53. doi: 10.1162/nol_a_00033 (PMC10158622; doi:10.1162/nol_a_00033)
Supplement: Supplementary file 7 [file nol-2-2-226-s007.pdf]

Supplementary material 7. Results of Correlations between Event-related field values and Coherence values at sensor and source level

Supplementary Table 7.1 Table of correlations between the squared response amplitudes from around 100 ms to syllables and coherence values collapsed across type and age from the delta and theta frequency for the left and right hemisphere separately

			Delta band	Theta band
Children	*Left peak (N=34)*	*Pearson Correlation*	0.148	0.181
		Sig. (2-tailed)	0.403	0.305
	Right peak (N=34)	*Pearson Correlation*	0.175	0.150
		Sig. (2-tailed)	0.323	0.398
Adults	*Left peak (N=19)*	*Pearson Correlation*	0.208	0.246
		Sig. (2-tailed)	0.393	0.310
	Right peak (N=19)	*Pearson Correlation*	0.032	-0.034
		Sig. (2-tailed)	0.895	0.891

Supplementary Table 7.2 Table of correlations between the squared response amplitudes for the pattern-checked N1m peaks to syllables and coherence values to words and sentences from the delta and theta frequency bands in the sensor level for the children and adults for the left and right hemisphere separately

				Delta band	Theta band
Children	*N1m peak left (N=10)*	*Words*	*Pearson Correlation*	0.112	-0.048
			Sig. (2-tailed)	0.757	0.894
		Sentence	*Pearson Correlation*	0.038	0.128
			Sig. (2-tailed)	0.917	0.724
	N1m peak right (N=13)	*Words*	*Pearson Correlation*	-0.534	0.187
			Sig. (2-tailed)	0.060	0.541
		Sentence	*Pearson Correlation*	0.236	0.334
			Sig. (2-tailed)	0.437	0.265
Adults	*N1m peak left (N=17)*	*Words*	*Pearson Correlation*	0.155	0.229
			Sig. (2-tailed)	0.553	0.376
		Sentence	Pearson Correlation	0.456	**0.506**
			Sig. (2-tailed)	0.066	**0.038**
	N1m peak right (N=17)	*Words*	*Pearson Correlation*	-0.221	0.094
			Sig. (2-tailed)	0.393	0.720
		Sentence	*Pearson Correlation*	-0.039	-0.241
			Sig. (2-tailed)	0.881	0.351

After correction for false discovery rate, none of the correlations remained significant.

Supplementary Table 7.3 Table of correlations between the squared response amplitudes for the pattern-checked N1m peaks to syllables and coherence values collapsed across type and age from the delta and theta frequency bands in the source level for the children and adults for the left and right hemisphere separately

			Delta band		Theta band	
			**Temporal ROI**	**Inferior-frontal ROI**	**Temporal ROI**	**Inferior-frontal ROI**
Children	*Left peak (N=34)*	*Pearson Correlation*	-0.120	-0.100	-0.001	0.041
		Sig. (2-tailed)	0.498	0.573	0.996	0.818
	Right peak (N=34)	*Pearson Correlation*	-0.064	0.024	0.026	0.092
		Sig. (2-tailed)	0.719	0.892	0.884	0.605
Adults	*Left peak (N=19)*	*Pearson Correlation*	0.220	0.411	0.051	0.083
		Sig. (2-tailed)	0.365	0.080	0.835	0.735
	Right peak (N=19)	*Pearson Correlation*	0.158	**0.461**	-0.036	0.270
		Sig. (2-tailed)	0.519	**0.047**	0.884	0.264

After correction for false discovery rate, none of the correlations remained significant.
